# Stem cell regulation: conserved pluripotency, transcriptional, and metabolic networks in animals and plants

**DOI:** 10.3389/fcell.2026.1779433

**Published:** 2026-08-14

**Authors:** Qiang Huang, Qi-Sheng Tang, Ming-Yue Qi, Xiao-Yu Yang, Hong-Qing Xie, Hui Guo, Xin-Yu Zhang

**Affiliations:** 1 Regenerative Medicine Research Center, The First People’s Hospital of Yunnan Province, The Affiliated Hospital of Kunming University of Science and Technology, Kunming, China; 2 Department of Stomatology, Shanghai General Hospital, Shanghai Jiaotong University School of Medicine, Shanghai, China

**Keywords:** animal stem cells, evolutionary conservation, metabolic regulation, plant stem cells, stemness maintenance, transcriptional regulation

## Abstract

Animal and plant stem cells have evolved lineage-specific regulatory networks over billions of years of independent evolution. However, their shared unicellular origin implies the preservation of shared ancestral regulatory modules and the repeated use of analogous molecular logic in stem cell control. Therefore, this narrative review aims to explore the molecular mechanisms common to animal and plant stem cells by focusing on three key aspects: stemness maintenance, transcriptional regulation, and metabolic control. In terms of stemness maintenance, the review highlights the pivotal functions of conserved proteins including breast cancer gene 1-associated really interesting new gene domain 1 (BARD1; animal protein)/AtROW1 (BARD1 plant homolog), retinoblastoma protein (RB; animal protein)/RETINOBLASTOMA-RELATED (RBR; RB plant homolog), and P-element induced wimpy testis (Piwi; animal protein)/ZWILLE (ZLL; Piwi plant homolog). In terms of transcriptional regulation, the review reveals the conserved functions of complexes and factors, such as Polycomb group PcG/Trithorax group proteins TrxG, switch/sucrose non-fermentable, MYC proto-oncogene (c-Myc; animal protein)/MYC (c-Myc plant homolog), Lin28/cold-shock domain protein 1, and Pumilio RNA-binding proteins in determining stem cell fate. In terms of metabolic regulation, the review delineates the convergent roles of threonine metabolism, target of rapamycin kinase signaling, glycogen synthase kinase (GSK3β; animal protein)/BRASSINOSTEROID-INSENSITIVE 2 (BIN2; GSK3β plant homolog), and nitric oxide signaling in orchestrating stem cell homeostasis by integrating nutrient and environmental signals. Despite vast differences in tissues and environments, the review findings suggest that stem cell systems across multicellular life forms share a common ancient molecular language for regulation. Overall, this review not only offers a fresh perspective on the evolution of stem cell mechanisms but also proposes testable hypotheses and conceptual frameworks for future cross-kingdom studies that may, in the long term, inform regenerative medicine research.

## 1 Introduction

After diverging from a shared unicellular ancestor over 1.6 billion years ago, animals and plants have retained similarities in their core transcriptional, translational, and post-transcriptional regulatory mechanisms (Meyerowitz, 2002; Olariu et al., 2017; De Mendoza and Sebé-Pedrós, 2019). Throughout their extensive independent evolution, animals and plants have evolved unique strategies for establishing and sustaining their stem cell pools (Matos and Bergmann, 2014). Stem cells are classically defined as undifferentiated cells with unlimited self-renewal and the ability to retain differentiation potential (Boiani and Schöler, 2005). In animals, the major stem cell types encompass embryonic stem cells (ESC) of embryonic origin and adult stem cells (tissue-resident stem cells in postnatal organisms) found within various tissues (Raff, 2003). In plants, stem cells are predominantly housed within apical and lateral meristems, which drive longitudinal growth and radial thickening, respectively (Sahoo et al., 2021). Stem cell homeostasis is governed by intricate transcriptional regulatory networks, which display considerable lineage-specific characteristics between animals and plants (Matos and Bergmann, 2014).

In plants, stem cell activity is governed by a finely tuned regulatory network. This network relies on auxin and cytokinin signaling. These signals converge on key downstream transcription factors, such as WUSCHEL (WUS) and SHOOT MERISTEMLESS (STM), thereby forming a critical regulatory nexus. (El-Showk et al., 2013; Ikeuchi et al., 2016; Rosspopoff et al., 2017; Bustillo-Avendaño et al., 2018; Wittmer and Heidstra, 2024). During embryogenesis and shoot apical meristem (SAM) establishment in Arabidopsis, auxin and cytokinin signaling pathways act in concert. They directly or indirectly regulate the expression of WUS and STM. (Aida et al., 1999; Gallois et al., 2002; Atta et al., 2009; Zhang et al., 2017; Maren et al., 2022; Yan et al., 2023; Wang et al., 2024). WUS is a key facilitator of somatic embryogenesis in many contexts, while its necessity can vary depending on the experimental system (Gordon et al., 2009; Su et al., 2009; Shpak and Uzair, 2025). In contrast, the primary function of STM is to inhibit cell differentiation, thereby maintaining an undifferentiated niche conducive for stem cell preservation (Greb and Lohmann, 2016).

Notably, at sites of organ primordia initiation, local auxin accumulation specifically represses STM expression. This alleviates its differentiation-inhibitory effect and allows stem cells to undergo organogenesis (Furutani et al., 2004; Heisler et al., 2005; Gordon et al., 2007). In addition, WUS and STM exhibit functional complementarity and engage in direct molecular interactions. In particular, these transcription factors co-activate the expression of the small peptide signaling molecule CLAVATA3 (CLV3), which acts as a negative feedback signal to restrict WUS expression and prevent stem cell over-proliferation (Brand et al., 2002; Su et al., 2020; Cao et al., 2023). CLV3 subsequently represses WUS expression to prevent stem cell over-proliferation (Cui et al., 2024; Wang et al., 2024). This results in the WUS-STM-CLV3 module, which constitutes a central regulatory negative feedback loop that precisely maintains stem cell homeostasis in shoot apical meristem. For the root apical meristem, conceptually analogous regulators, such as WOX5 and root-specific CLE peptides participate in tissue-specific regulatory mechanisms. (Su et al., 2020). In the RAM, WOX5 protein moves from the quiescent center (QC) into adjacent columella stem cells (CSCs), where it directly represses the transcription factor gene CDF4 by recruiting TPL/TPR co-repressors and the histone deacetylase HDA19, consequently inducing histone deacetylation at the CDF4 regulatory region (Pi et al., 2015).

Throughout these processes, auxin and cytokinin exhibit context-dependent antagonistic and synergistic interactions (Dello Ioio et al., 2008; Moubayidin et al., 2009). For instance, in the root meristem, auxin and cytokinin often act antagonistically to balance cell division and differentiation (Ikeuchi et al., 2016). In the context of stem cell reprogramming and regeneration, however, cytokinin plays a crucial role. For example, within callus tissues, elevated cytokinin signaling induces ectopic WUS expression, which in turn triggers shoot regeneration (Gordon et al., 2009). This insight explains the mechanism by which ectopic expression of WUS and STM alone reprograms differentiated cells into stem cells, thus enabling *de novo* formation of functional meristems within differentiated tissues.

In the root, PLETHORA (PLT) transcription factors form a dosage-dependent gradient that specifies stem cell identity (Galinha et al., 2007; Mähönen et al., 2014), while the auxin-responsive repressor SHY2/IAA3 and type-B ARABIDOPSIS RESPONSE REGULATORS (ARRs) mediate reciprocal auxin–cytokinin crosstalk to balance cell division and differentiation (Dello Ioio et al., 2008). Local auxin biosynthesis by YUCCA (YUC) flavin monooxygenases is essential for maintaining auxin maxima in both root and shoot stem cell niches (Zhao, 2018). In the shoot apical meristem, KNOX transcription factors integrate cytokinin and gibberellin signals to sustain stem cell organization (Jasinski et al., 2005; Bolduc and Hake, 2009).

Somatic embryogenesis is a remarkable reprogramming process. Key transcription factors are reactivated to specify the apical stem cell niches in the newly formed embryo. Similarly, metabolic pathways exhibit conserved functions in both meristem maintenance and the induction of embryogenic competence. (Brand et al., 2002; Gallois et al., 2002; Lenhard et al., 2002).

In animals, the wingless-related integration site (Wnt) and Notch signaling pathways engage in complex synergistic interactions and crosstalk. Collectively, these pathways constitute a core regulatory network that governs stem cell pluripotency, self-renewal, and fate determination. Individually, both the Wnt and Notch pathways play pivotal roles in modulating and sustaining stem cell activity (Lampreia et al., 2017; Mills et al., 2017). Specifically, the canonical Wnt/β-catenin signaling cascade participates in stem cell self-renewal and progenitor cell proliferation or differentiation (Van Camp et al., 2014). In the absence of Wnt ligands, glycogen synthase kinase 3β (GSK3β) phosphorylates β-catenin, which prepares it for ubiquitination and subsequent degradation and prevents its entry into the nucleus (Latres et al., 1999). However, upon Wnt ligand binding to the frizzled receptor and its low-density lipoprotein receptor-related protein (LRP) co-receptor, the LRP receptor is phosphorylated by GSK3β. This event triggers the dissociation of β-catenin from the Axin-containing destruction complex, thereby enabling its nuclear translocation where it activates target gene transcription to regulate stem cell homeostasis (Metcalfe et al., 2010; Yang et al., 2020; Batista et al., 2021). In addition, non-canonical Wnt pathways, including the Wnt/receptor tyrosine kinase and Wnt/Ca^2+^ cascades, similarly contribute to stem cell maintenance, directed cell migration, or the suppression of canonical Wnt signaling (Qin et al., 2015; Wang et al., 2015; Katoh, 2017).

Correspondingly, activation of the Notch pathway promotes self-renewal, metastasis, and survival in cancer stem cells, which concurrently suppresses cell death (Bisht et al., 2022; Shi et al., 2024). Within the canonical Notch pathway, ligand-activated Notch receptors undergo proteolytic cleavage, and the resulting intracellular domain translocates to the nucleus. In the nucleus, the domain binds to C-promoter-binding factor 1/suppressor of hairless/Lin-12 and Glp-1, displaces co-repressors, and recruits co-activators to initiate transcription of target genes (Shi et al., 2024). In contrast, non-canonical Notch signaling functions by post-translationally modulating components of the Wnt/β-catenin pathway (Andersen et al., 2012). Furthermore, non-canonical Notch interacts with phosphatase and tensin homolog-induced kinase 1, leading to activation of the mechanistic target of rapamycin (mTOR) complex 2/protein kinase B (AKT) signaling axis, which sustains brain tumor stem cell populations (Perumalsamy et al., 2009; Lee et al., 2013).

Beyond these individual roles, the Wnt and Notch pathways are interconnected through complex crosstalk. The Wnt pathway directly upregulates the expression of Jagged 1, a key ligand of the Notch pathway, thereby potentiating Notch signal activation (Katoh and Katoh, 2006). Conversely, Notch signaling is capable of modulating the Wnt/β-catenin signaling pathway, thereby influencing Wnt pathway activity (Kwon et al., 2011; Andersen et al., 2012). Collectively, the Wnt and Notch signaling cascades constitute a core stem cell signaling network (Katoh, 2017), with their interplay exerting decisive regulatory control over animal stem cell fate (Batista et al., 2021). Hence, these two pathways not only operate via unique transduction mechanisms but also collaboratively determine stem cell fate through multi-faceted interactions.

Considering that animal and plant stem cells share a unicellular origin, this narrative review aims to discuss the molecular mechanisms common to animal and plant stem cells. The review focuses on three key aspects: stemness maintenance, transcriptional regulation, and metabolic control.

This review is conceived as a narrative review rather than a systematic review. Given the broad, interdisciplinary nature of the topic—spanning plant and animal stem cell biology, evolutionary developmental biology, and comparative regulatory network analysis—our aim is to synthesize and interpret key findings from diverse fields to reveal overarching conceptual principles. Accordingly, we have adopted a thematic, integrative approach to compare and contrast the conserved regulatory mechanisms across kingdoms.

## 2 Shared mechanisms underpinning plant and animal stem cell maintenance

Plant and animal stem cells exhibit parallels in regulating the size of their stem cell pools. In both systems, stem cell maintenance constitutes a core function and is orchestrated by a multitude of factors.

In animals, the tumor suppressor protein BRCA1-associated RING domain 1 (BARD1) forms a functional heterodimer with BRCA1 through its RING finger domain. This complex plays a critical role in preserving genomic stability by facilitating DNA double-strand break repair. Although this function is essential for the long-term integrity of rapidly dividing stem cells (e.g., germline and ESCs), it is not specific to stem cells but rather reflects a universal DNA damage response (Elledge and Amon, 2002; Shahin et al., 2023; Li et al., 2025). In striking contrast, the plant functional homolog of BARD1, AtROW1, has evolved distinct roles directly governing stem cell fate. AtROW1 represses WUS transcription, thereby restricting its expression domain to the organizing center of the shoot apical meristem (SAM) and directly contributing to stem cell homeostasis (Reidt et al., 2006; Jiao et al., 2016). Notably, mutation of AtROW1 results in the formation of tumor-like root cell masses, phenocopying aspects of uncontrolled proliferation (Han et al., 2008). Furthermore, AtROW1 also represses WOX5 in the root apical meristem by binding H3K4me3 and recruiting PRC2 to deposit H3K27me3 at the WOX5 promoter, thereby safeguarding the root stem cell niche. Phylogenetic analysis confirms that the components of the BARD1/BRCA1 complex share a common eukaryotic ancestor that predates the plant–animal divergence (Jiao et al., 2016). Despite the clear homology of the core protein domains, their functions have diverged, providing a striking example of lineage-specific neo-functionalization of an ancestral DNA repair module. The BARD1/AtROW1 module exemplifies how an evolutionarily ancient protein complex has been co-opted into kingdom-specific regulatory networks to maintain stem cell homeostasis.

AtROW1 also represses WOX5 transcription in the root apical meristem by binding H3K4me3 and recruiting PRC2 to deposit histone H3 lysine 27 (H3K27me3) at the WOX5 promoter. Loss of ROW1 causes ectopic WOX5 expression, leading to quiescent centre failure, stem cell niche disorganization, and root growth/gravitropism defects. Genetic epistasis confirms WOX5 acts downstream of ROW1, as the wox5-1/row1-3 double mutant phenocopies wox5-1. Thus, ROW1 safeguards both shoot and root stem cell niches by restricting WUS and WOX5 expression to their organizing centres, revealing a conserved regulatory logic across meristems (Zhang et al., 2015).

The RETINOBLASTOMA-RELATED (RBR) protein in plants and its animal homolog retinoblastoma (RB) represent another conserved stem cell regulator across kingdoms. The central interaction between RBR/RB and eukaryotic 2 transcription factor (E2F) is universal among eukaryotes. By forming complexes with E2F/dimerization partner family members, RBR/RB represses the expression of genes necessary for S-phase and mitosis entry, thus arresting the cell cycle at the G1/S and G2/M checkpoints (Gutzat et al., 2011; Desvoyes and Gutierrez, 2020; Zamora-Zaragoza et al., 2024). In animals, RB is a master regulator of stem cell proliferation, differentiation, and survival (Gutzat et al., 2012; Sage, 2012). *RB* mutation disrupts cell cycle control, leading to uncontrolled proliferation and tumor formation (Zhou et al., 2024). A key mechanism in mammals involves RB recruiting Polycomb repressive complex (PRC) 2 to catalyze trimethylation of H3K27me3 at the promoter of the cell cycle inhibitor p16. This epigenetic mark facilitates subsequent recruitment of PRC1-related complexes, resulting in stable silencing of p16. This pathway critically influences the cell cycle to regulate stem cell proliferation and self-renewal (Kotake et al., 2007). Similarly, in *Arabidopsis thaliana*, RBR facilitates the silencing of late-embryogenesis genes by activating PRC2-mediated formation of H3K27me3 (Gutzat et al., 2011). RNA interference-mediated knockdown of *RBR* disrupts proper cell division and maintenance in every stem cell niche (Borghi et al., 2010). While the core cell-cycle regulatory function is deeply conserved, the recruitment of this module to specifically silence stem cell regulators (e.g., WUS in plants, p16 in animals) appears to represent a convergent evolutionary strategy. Moreover, elevated RBR expression drives stem cell differentiation, whereas its downregulation results in the expansion of the stem cell pool (Gutzat et al., 2012). RBR also directly represses WUS transcription to prevent ectopic stem cell activity (Zhao et al., 2017). In the Arabidopsis root meristem, where WUS is not expressed, RBR maintains the stem cell niche by controlling cell cycle progression at the G1/S and G2/M checkpoints via the E2F/DP pathway, and by indirectly modulating the expression of the WUS homolog WOX5 through the SCARECROW-SHORTROOT protein network (Wildwater et al., 2005; Desvoyes and Gutierrez, 2020).

The P-element induced wimpy testis (Piwi) protein, a member of the argonaute family, is essential for stem cell self-renewal and demonstrates remarkable functional conservation across animals and plants (Benfey, 1999; Rodríguez-Leal et al., 2016; Stöhr et al., 2019). In *Drosophila,* Piwi is crucial for maintaining germline stem cells (GSC) in an undifferentiated state (Cox et al., 1998; Rojas-Ríos and Simonelig, 2018). The protein also safeguards GSC genomic integrity by silencing transposable elements (TE) via its associated Piwi-interacting RNAs. Furthermore, Piwi promotes GSC self-renewal by activating the translation of mRNAs for glycolytic enzymes, such as enolase, thus modulating the metabolic balance between self-renewal and differentiation (Rojas-Ríos et al., 2024). Moreover, Piwi fine-tunes gene expression by inhibiting the binding of PRC2 to specific targets, thereby reducing repressive H3K27me3 marks and preventing the over-silencing of genes required for GSC maintenance (Claro-Linares and Rojas-Ríos, 2025). Mutations in Piwi genes result in genomic instability within germline stem cells, ultimately leading to sterility in both *Drosophila* and mammals (Stolyarenko, 2020; Wang et al., 2023). In *A. thaliana*, the Piwi homolog ZWILLE (ZLL) is similarly pivotal for stem cell homeostasis and maintains stem cells in an undifferentiated state. ZLL also regulates the spatial expression pattern of the *STM* gene during late embryogenesis to inhibit differentiation (Moussian et al., 1998). Furthermore, ZLL contributes to the maintenance of plant ESCs by potentiating WUS signaling (Tucker et al., 2008).

**FIGURE 1:**
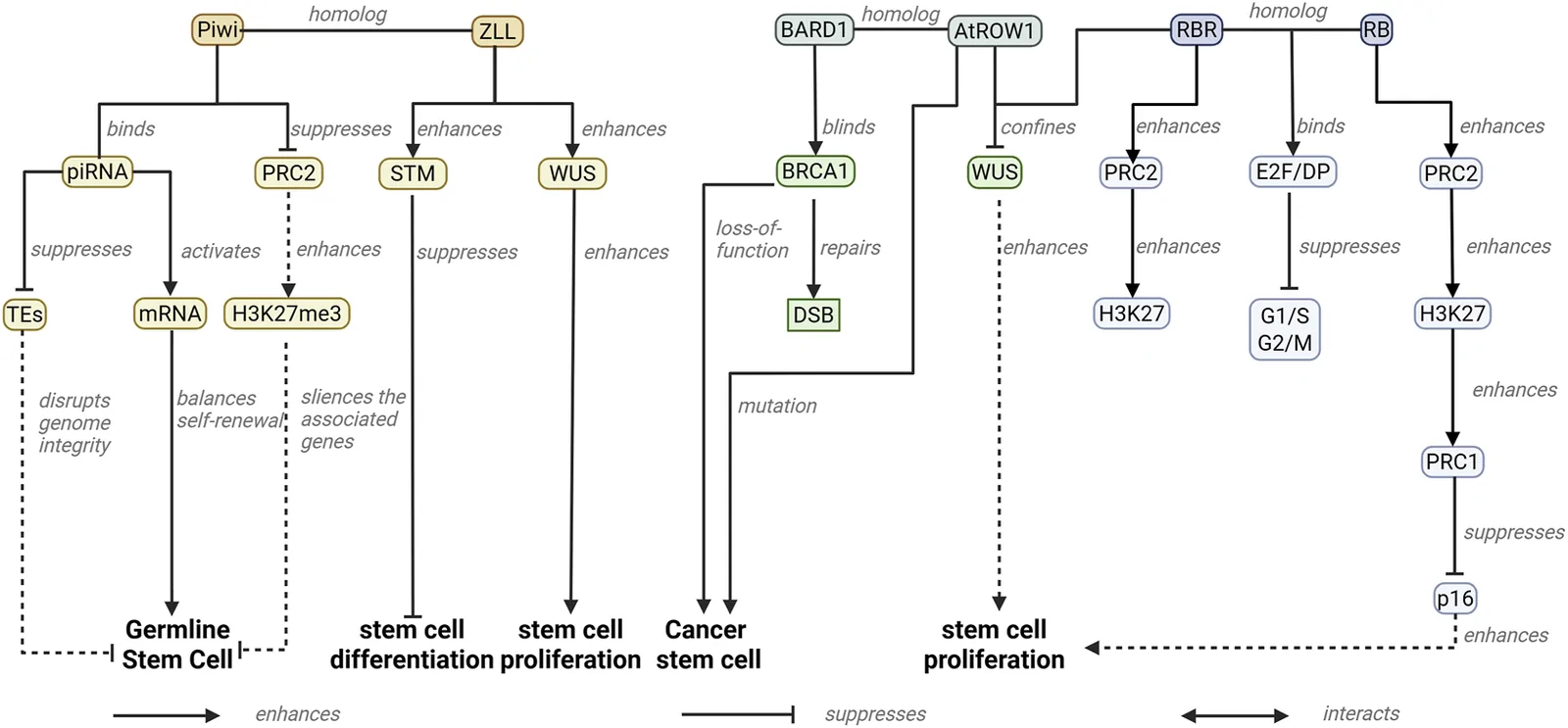
Shared mechanisms underlying stem cell maintenance in plants and animals. The BRCA1/BARD1 complex in animals and its plant homolog AtROW1 maintain stem cell integrity through DNA damage repair and transcriptional repression of key stem cell factors, such as WUS, respectively. The RB/RBR-E2F module controls the G1/S cell cycle checkpoint in both kingdoms, with plant RBR directly repressing WUS expression. The conserved Piwi/ZLL module, part of the argonaute protein family, is essential for stem cell self-renewal. The module protects genomic integrity by silencing TEs and fine-tunes stem cell fate by regulating the expression of core transcription factors, such as WUS and STM. Abbreviations: BRCA1, breast cancer gene 1; BARD1, BRCA1-associated really interesting new gene domain 1; AtROW1, BARD1 plant homolog; WUS, WUSCHEL; RB, retinoblastoma protein; RBR, RETINOBLASTOMA-RELATED (RB plant homolog); E2F, eukaryotic 2 transcription factor; Piwi, P-element induced wimpy testis; ZLL, ZWILLE (Piwi plant homolog); TE, transposable element; STM, STM, SHOOT MERISTEMLESS; piRNA, Piwi-interacting RNA; PRC, Polycomb repressive complex; E2F, eukaryotic 2 transcription factor; DP, dimerization partner; H3K27me3, trimethylated histone H3 lysine 27; DSB, DNA double-strand break; H3K27; histone H3 lysine 27.

Altogether, the mechanisms underlying stemness maintenance in plant and animal stem cells demonstrate deep evolutionary conservation at multiple regulatory layers (Figure 1). The shared regulatory modules (BARD1/AtROW1, RBR/RB, and Piwi proteins) highlight fundamental commonalities within the core regulatory networks governing stem cell maintenance across kingdoms.

## 3 Convergent mechanisms in transcriptional regulation of plant and animal stem cells

Although distinctions exist in the transcriptional regulatory networks of plant and animal stem cells, several key transcription factors and regulatory mechanisms are shared and operative in both systems (Olariu et al., 2017). The genomes of both plants and animals employ conserved histone families to assemble euchromatin and heterochromatin structures. This relies on an evolutionarily conserved suite of core histones that undergo essential post-translational modifications (Birnbaum and Sánchez Alvarado, 2008). Consequently, plants and animals possess analogous fundamental mechanisms for chromatin structure regulation (Costa and Shaw, 2007).

Key transcription factors, such as WUS and STM, contain a homeodomain structurally related to that of animal homeobox (HOX) proteins, which share homeodomain motif. (Laux et al., 1996). The homeodomain, first identified in *Drosophila*, is a conserved DNA-binding motif found in both plants and animals (Schneuwly et al., 1986). These factors govern fundamental cellular processes including proliferation, differentiation, and apoptosis, thereby playing pivotal roles in shaping tissues and organs during embryogenesis (Ye et al., 2011).

In *Drosophila*, the stable repression of homeotic (HOX) gene expression is mediated by Polycomb Group (PcG) proteins (Schuettengruber et al., 2017), whereas their active state is maintained by Trithorax group (TrxG) proteins (Simon and Tamkun, 2002). Both PcG and TrxG complexes are evolutionarily conserved chromatin regulators in plants and animals. PcG and TrxG typically function antagonistically to regulate transcription (Pien and Grossniklaus, 2007; Schuettengruber et al., 2017; Xu et al., 2024). TrxG complexes oppose PcG-mediated silencing, often by facilitating switch/sucrose non-fermentable (SWI/SNF)-mediated chromatin remodeling to activate gene expression (Kadoch et al., 2017; Stanton et al., 2017). The SWI/SNF family represents a major class of evolutionarily conserved chromatin remodelers in yeasts, animals, and plants, with critical functions in stem cell fate determination across kingdoms (Muchardt and Yaniv, 1999; Lin et al., 2023). The adenosine triphosphatase (ATPase) BRAHMA (BRM) is a core catalytic subunit of the SWI/SNF complex (Lin et al., 2021). BRM also functions as a TrxG protein that is capable of antagonizing PcG-mediated repression in *Drosophila* and mammals (Li et al., 2015). In mice, *BRM* null mutation causes aberrant proliferation and functional impairment of muscle stem cells, thereby compromising post-injury muscle regeneration and delaying new myofiber formation (Albini et al., 2015).

In *Arabidopsis*, *BRM* null mutation disrupts root stem cell maintenance, leading to reduced meristem activity and stunted root growth (Li et al., 2015; Wrona et al., 2024). Within the SAM, the BRM-related ATPase SPLAYD (SYD) is recruited to the *WUS* promoter to directly activate its transcription, thereby participating in the regulation of stem cells, including those giving rise to reproductive structures (Kwon et al., 2005).

Moreover, in animals, the somatic reprogramming factor MYC protooncogene (c-Myc) recruits SWI/SNF complexes and histone acetyltransferases to drive chromatin alterations (Matos and Bergmann, 2014). Furthermore, c-Myc regulates a broad spectrum of stem cells, such as neural, hematopoietic, and cardiac stem cells. The plant homolog of c-Myc (MYC) belongs to the same large basic helix-loop-helix (bHLH) transcription factor family (Abe et al., 1997; Matos and Bergmann, 2014). During plant development, specific c-Myc homologs belonging to the bHLH transcription factor family (e.g., AtMYC2 in Arabidopsis) are negatively regulated by WUS and function in regulating meristematic stem cells (Yang et al., 2012; Makkena and Lamb, 2013; Schuster et al., 2014). In animals, c-Myc regulates a broad spectrum of stem cells, such as neural, hematopoietic, and cardiac stem cells (Conway et al., 2010; Curtis et al., 2012; Lai et al., 2013). c-Myc plays various roles in pluripotent stem cells, including the promotion of somatic cell reprogramming to pluripotency, the regulation of cell competition and the control of embryonic diapause. A specific enhancer cluster regulates Myc transcription in early mouse embryos and embryonic stem cells. This reveals the modular regulatory mechanism of Myc expression in different pluripotency states, providing new insights into the regulation of Myc in stem cells (Li-Bao et al., 2024).

PcG proteins contribute to pluripotency by repressing lineage-specific genes in ESCs, whereas genes required for ESC self-renewal and the undifferentiated state escape repression and remain actively transcribed (Schuettengruber et al., 2017). PcG proteins also silence chromatin in stem cells via histone modifications, including methylation and deacetylation (Otte and Kwaks, 2003). PcG functions through two primary complexes: PRC1 and PRC2. PRC2 catalyzes the repressive H3K27me3 mark, which initiates chromatin silencing. Thereafter, PRC1 binds to H3K27me3 and compresses the chromatin, thereby working synergistically to repress PcG target genes (Li et al., 2015). In *Arabidopsis,* PRC2-like complexes repress genes, such as *AGAMOUS* and *STM* via H3K27me3 deposition (Schubert et al., 2006). Mutations in PRC2 subunit genes reverse the repression of ectopic growth in vegetative tissues, resulting in the formation of callus-like or somatic embryo structures (Wittmer and Heidstra, 2024). Furthermore, dysfunction of the PRC2 subunits CURLY LEAF (CLF) or SWINGER (SWN) causes derepression (ectopic expression) of WUS, which subsequently leads to disintegration of the SAM or embryonic lethality (Schubert et al., 2006; Chen et al., 2010; Zhou et al., 2018). Thus, PcG and TrxG proteins are pivotal transcriptional regulators of stem cell fate.

The cold-shock domain protein 1 of *Physcomitrella patens* (PpCSP1) is a functional homolog of the animal pluripotency factor Lin28. PpCSP1 shares both functional and sequence homology with Lin28 (Li C. et al., 2017), which suggests conserved mechanisms. Furthermore, both are RNA-binding proteins that modulate mRNA metabolism, including maturation, stability, and translation, in the cytoplasm (Tanabe et al., 2013).

PpCSP1/Lin28 is primarily associated with reprogramming, where it facilitates the conversion of somatic cells into induced pluripotent stem cells by promoting mesenchymal-to-epithelial transition and activating pluripotency networks. In animals, Lin28, in combination with other core factors (octamer-binding transcription factor 4, SRY-box transcription factor 2, Nanog homeobox), reprograms human fibroblasts into induced pluripotent stem cells (Yu et al., 2007). Similarly, PpCSP1 reprograms differentiated leaf cells into shoot apical stem cells. Therefore, PpCSP1 and Lin28 both function as key cellular reprogramming regulators (Li C. et al., 2017; Ishikawa and Hasebe, 2022). Moreover, upon physical wounding of leaves, PpCSP1 is induced at the injury site by stem cell-inducing factors, where it potentiates the reprogramming of wounded cells (Li C. et al., 2017). PpCSP1 also function in a wound-independent manner to re-establish meristems (Ishikawa et al., 2019). Furthermore, loss-of-function mutations in *PpCSP1* inhibit shoot apical stem cell growth, leading to delayed apical development (Ishikawa and Hasebe, 2022).

Pumilio (Pum) proteins represent a family of evolutionarily conserved RNA-binding proteins ubiquitous across eukaryotes, including yeast, plants, and animals (Spassov and Jurecic, 2003), in which they function as key post-transcriptional regulators (Phan et al., 2025). Functionally, Pum and its orthologs are vital regulators of development, cell proliferation, and stem cell differentiation across eukaryotes (Goldstrohm et al., 2018). Pumilio is predominantly involved in stemness maintenance, and sustains self-renewal and prevents differentiation in established stem cell populations. In *Drosophila*, Pum governs germline stem cell proliferation, embryogenesis, and neural function (Arvola et al., 2017). In mice, double mutation of Pum genes severely compromises ESC self-renewal and differentiation, causing developmental retardation and lethality by embryonic day 8.5 (Uyhazi et al., 2020). In plants, Pum homologs contribute to shoot apical stem cell maintenance (Francischini and Quaggio, 2009). Structurally, Pum proteins feature a highly conserved RNA-binding domain made of eight tandem repeats of a triple α-helical motif. Within each repeat, specific amino acids mediate sequence-specific recognition and binding of individual RNA bases (Weidmann et al., 2016; Haugen et al., 2024). Mechanistically, Pum functions as a repressor. It binds specific mRNAs via its recognition element, leading to accelerated mRNA decay and/or inhibition of translation, thereby suppressing protein output. (Uyhazi et al., 2020). Moreover, Pum-mediated translational inhibition involves the disruption of poly(A) tail-binding protein activity (Arvola et al., 2020). Pum proteins are evolutionarily conserved post-transcriptional regulators. They play a crucial role in determining stem cell fate across diverse biological contexts.

**FIGURE 2:**
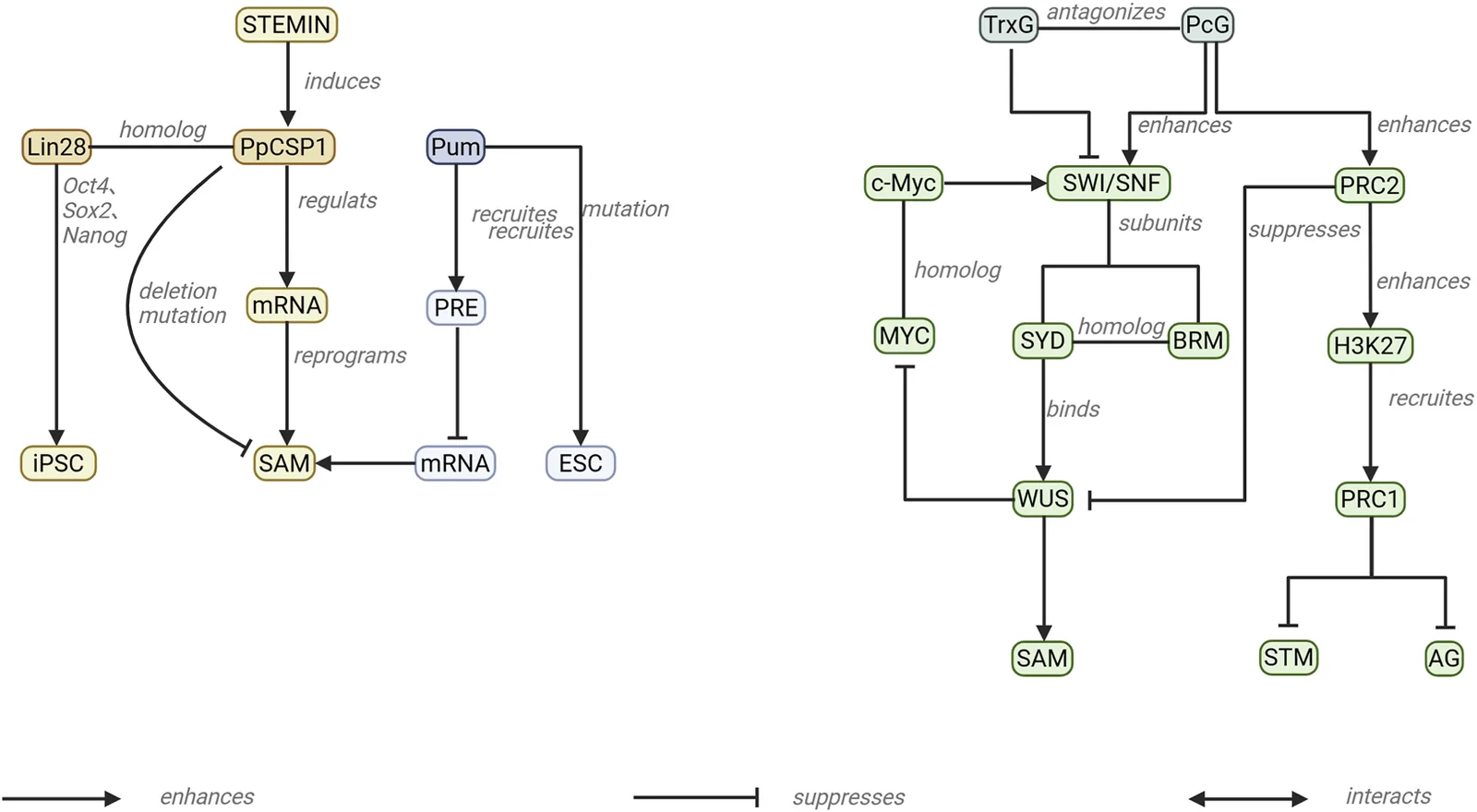
Similar transcriptional regulatory mechanisms in plant and animal stem cells. The antagonistic Polycomb group (PcG) and Trithorax group (TrxG) complexes maintain repressive (H3K27me3) or active chromatin states at target loci. PcG represses genes, such as WUS and STM in plants and lineage-specific genes in animals, whereas TrxG counteracts this silencing. The SWI/SNF chromatin remodeling complex, a TrxG component, is recruited by factors, such as c-Myc/MYC to activate transcription. In plants, SWI/SNF ATPases (e.g., SYD) directly bind to the WUS promoter to activate its expression. Conserved RNA-binding proteins, including Lin28/PpCSP1 and Pum, regulate stem cell fate post-transcriptionally by modulating mRNA metabolism, thereby contributing to cellular reprogramming and maintenance. Abbreviation: PcG, Polycomb group; TrxG, Trithorax group; H3K27me3, trimethylated histone H3 lysine 27; WUS, WUSCHEL; STM, SHOOT MERISTEMLESS; SWI/SNF, switch/sucrose non-fermentable; c-Myc, MYC protooncogene; MYC, c-Myc plant homolog; ATPase, adenosine triphosphatase; SYD, SPLAYD; PpCSP1, cold-shock domain protein 1; Pum, Pumilio; STEMIN, stem cell-inducing factor; iPSC, induced pluripotent stem cell; SAM, shoot apical meristem; PRE, Pum recognition element; ESC, embryonic stem cell; PRC, Polycomb repressive complex; BRM, BRAHMA; H3K27, histone H3 lysine 27; AG, AGAMOUS.

Altogether, the transcriptional regulation of plant and animal stem cells involves a suite of key factors that are highly convergent in both function and mechanism. These shared factors include the antagonistic PcG/TrxG systems that maintain transcriptional states, reprogramming facilitators, such as PpCSP1, and post-transcriptional regulators, such as Pum. All these factors reveal the fundamental commonalities in stem cell fate determination. This emphasizes that multicellular life shares a deep and ancient molecular language for regulating stem cell fate (Figure 2).

## 4 Convergent metabolic regulation in plant and animal stem cells

Plant and animal stem cells share analogous metabolic dependencies in their metabolic dependencies during development. In particular, threonine metabolism emerges as a critical factor convergently required for stem cell maintenance in both kingdoms (Reyes-Hernández et al., 2019). In cultured mouse embryonic stem cells (mESCs), threonine deprivation blocks proliferation and drives differentiation (Wang et al., 2009). Furthermore, threonine catabolism contributes to the one-carbon (1C) metabolic network, and this threonine-1C metabolism axis is believed to regulate mESC function potentially through modulating histone H3 lysine 4 (H3K4) methylation (Sahoo et al., 2021; Niedzialkowska et al., 2022). In this specific experimental system, threonine also facilitates mTOR signaling, partly by upregulating c-Myc expression (Ryu and Han, 2011). While these findings highlight the importance of threonine in mESCs, direct evidence supporting an equivalent universal requirement across all mammalian stem cell types remains to be established.

Target of rapamycin (TOR) is a highly conserved serine/threonine kinase with essential roles in both animal and plant stem cells. In animal models, mTOR acts as a master regulator that integrates diverse inputs, including nutrients, energy status, hormones, and environmental cues, to coordinate cell growth and cycle progression (Fingar and Blenis, 2004). In plant models, particularly in *Arabidopsis thaliana*, TOR kinase regulates root and shoot growth and is also involved in callus formation (Pfeiffer et al., 2016; Lee and Seo, 2017; Li X. et al., 2017). Notably, studies in *Arabidopsis* have demonstrated that TOR kinase plays a core role in the light-dependent activation of WUS and the subsequent reactivation of stem cells in the shoot apical meristem (Pfeiffer et al., 2016).

In animals, the mTOR signaling pathway is potently activated by Akt, which amplifies the signal to promote cell proliferation (Limon and Fruman, 2012). A key downstream action of Akt is the direct phosphorylation and inhibition of GSK3β, a negative regulator of stem cell renewal in animals (Hermida et al., 2017). Accordingly, GSK3β inhibitors can substitute for exogenous transcription factors during the chemical reprogramming of mammalian somatic cells (Zhu et al., 2010). The functional homolog of GSK3β in plants is BRASSINOSTEROID-INSENSITIVE 2 (BIN2). Strikingly, pharmacological inhibition of BIN2 also induces embryonic transitions in plant somatic cells *in vitro* (Berenguer et al., 2021). Furthermore, Furthermore, in *Arabidopsis*, TOR regulates the phosphorylation of ribosomal protein S6 kinase β2 (S6K2), which interacts with and directly phosphorylates BIN2, linking TOR signaling to this conserved regulatory node for plant growth control (Xiong et al., 2017).

Additional metabolic factors are crucial for stem cell fate decisions in plants, a key example being nitric oxide (NO). In mammalian systems, NO is a highly reactive, short-lived gaseous molecule synthesized by nitric oxide synthase (NOS) enzymes via the oxidation of L-arginine to L-citrulline (Marletta, 1994; Caballano-Infantes et al., 2022). Low NO concentrations delay differentiation and promote the survival of ESCs (Tejedo et al., 2010). Moreover, NO regulates stem cell differentiation through both cyclic guanosine monophosphate (cGMP)-dependent and cGMP-independent signaling pathways (Mujoo et al., 2011). Therefore, attaining precise thresholds of NO and cGMP is critical for normal embryogenesis, as deviations can cause developmental arrest or apoptosis in mouse embryos. For instance, inhibiting NO production impedes the progression from the two-cell to the four-cell stage. However, excessive NO levels also disrupt embryonic development (Tranguch et al., 2003). In plant models, NO is involved in a wide array of processes, including seed germination, root development, and flowering (Beligni and Lamattina, 2000; He et al., 2004; Yu et al., 2014). Plants lack canonical NOS enzymes; therefore, a major source of NO in land plants is the reduction of nitrite catalyzed by nitrate reductase (Fancy et al., 2017).

Analogous to its role in mammalian embryonic development, NO levels are linked to plant embryonic development. In *Arabidopsis*, reduced NO concentrations inhibit primary root meristem growth (Sanz et al., 2014). Furthermore, in alfalfa cell cultures, NO activates cell division and embryo formation, potentially through interactions with factors, such as auxin (Otvös et al., 2005). Moreover, in the *Arabidopsis* shoot apex, NO regulates stem cells by limiting ARGONAUTE 4 (AGO4) accumulation in the shoot apex, thereby modulating its activity. Given the physical interaction between AGO4 and WUS, NO-mediated restriction of this interaction represses WUS expression, thereby fine-tuning shoot apical stem cell homeostasis (Zeng et al., 2023). In line with these developmental roles, the manipulation of metabolic pathways and associated signaling nodes has been shown to directly influence somatic embryogenesis efficiency. For instance, altering TOR activity or endogenous NO levels during tissue culture is sufficient to modulate the induction and progression of SE in multiple plant species, underscoring that the metabolic control of stem cell fate can be harnessed to enhance regenerative outcomes. These metabolic similarities likely represent functional convergence driven by the universal need to balance energy production, biosynthesis, and epigenetic regulation in rapidly dividing cells, rather than lineal inheritance of a specific stem cell pathway.

**FIGURE 3:**
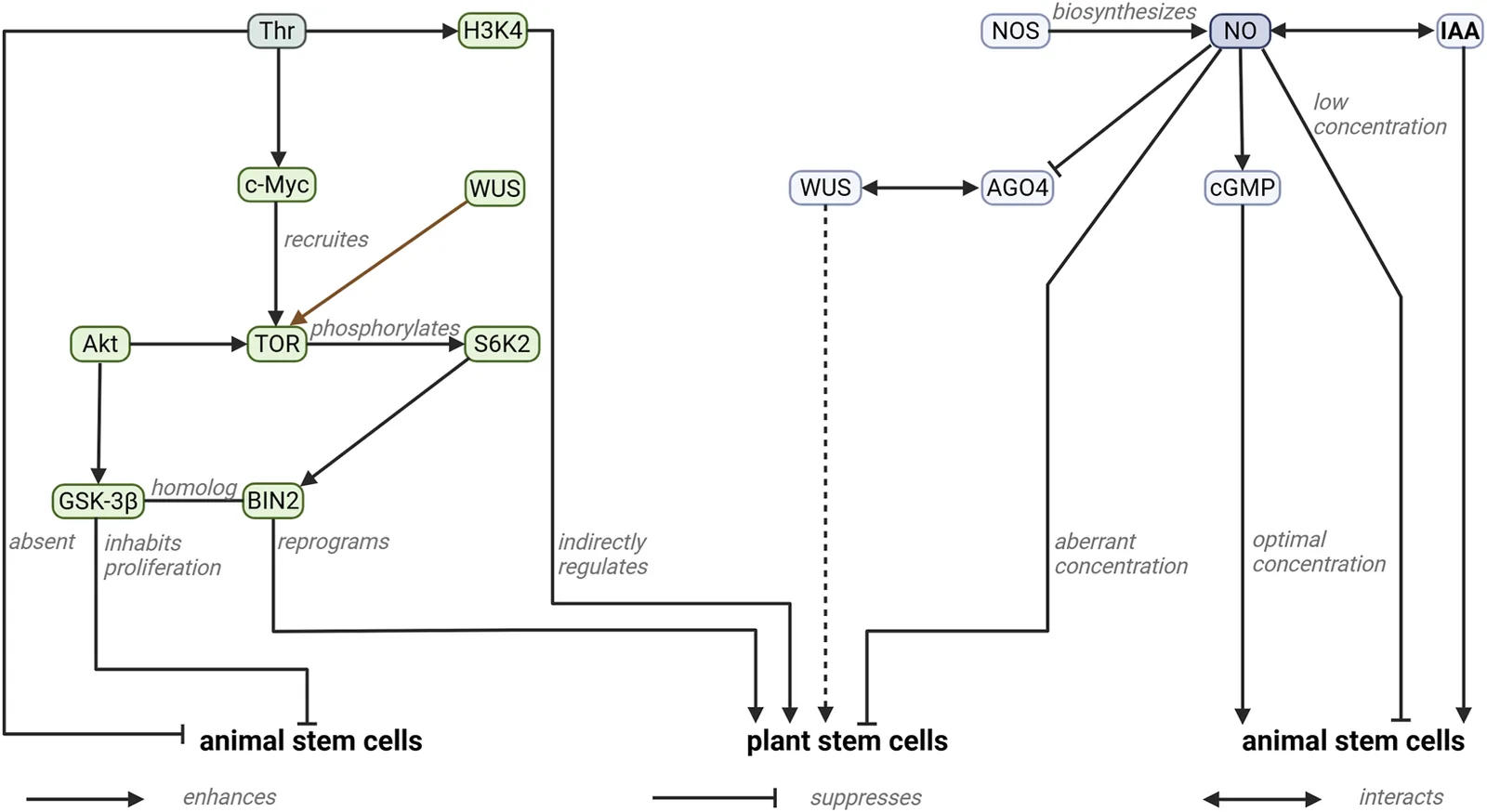
Common metabolic regulators in plant and animal stem cells. Threonine metabolism fuels one-carbon cycles and influences histone methylation (e.g., H3K4me3) and c-Myc/MYC expression, which are critical for stem cell maintenance. The central kinase TOR/mTOR integrates nutrient, energy, and environmental signals. In plants, TOR activation leads to WUS overexpression and stem cell proliferation. The conserved GSK3β/BIN2 kinase acts as a regulatory node; its inhibition promotes stem cell renewal and somatic cell reprogramming. The gaseous molecule NO fine-tunes stem cell activity in a concentration-dependent manner. In plants, NO represses WUS by disrupting the AGO4-WUS interaction. Abbreviations: Thr, threonine; H3K4me3; trimethylated histone H3 lysine 4, c-Myc, MYC protooncogene; MYC, c-Myc plant homolog; TOR, target of rapamycin; mTOR, mechanistic TOR; WUS, WUSCHEL; GSK3β, glycogen synthase kinase; BIN2, BRASSINOSTEROID-INSENSITIVE 2 (GSK3β plant homolog); NO, nitric oxide; AGO4, ARGONAUTE 4; H3K4; histone H3 lysine 4; Akt, protein kinase B; S6K2, ribosomal protein S6 kinase β2; NOS, NO synthase; IAA, indole-3-acetic acid; cGMP, cyclic guanosine monophosphate.

Overall, the metabolic regulation of plant and animal stem cells have profound convergences. Specifically in terms of the conserved roles of central nutrient-sensing pathways (threonine metabolism, TOR, GSK-3β/BIN2) and the nuanced regulation by signaling molecules (NO). These elements form an evolutionarily conserved metabolic network that precisely instructs stem cell fate through integrated energy and signaling cues (Figure 3).

## 5 Summary and perspectives

This review compares and underscores the convergent mechanisms governing stem cell maintenance, transcriptional regulation, and metabolic control in plants and animals. A comprehensive summary of these conserved regulatory modules is provided in Table 1. Beyond cataloging shared modules, this review reveals that key plant stem cell regulators, notably the homeodomain transcription factors WUS and STM, are recurrently embedded within these evolutionarily conserved networks. This recurrent integration suggests that the regulatory principles embodied by WUS and STM may inform current understanding of cell fate determination in animal stem cells.

**TABLE 1:** Conserved regulatory modules in plant and animal stem cells.

Regulatory module (homologs)	Animal evidence and function	Plant evidence and function	Comparison type
BARD1/AtROW1	BARD1 forms heterodimer with BRCA1; involved in DNA double-strand break repair in germline and ESCs (Elledge and Amon, 2002; Shahin et al., 2023)	AtROW1 represses WUS and WOX5 transcription by binding H3K4me3 and recruiting PRC2 for H3K27me3 deposition; restricts stem cell niche boundaries (Han et al., 2008; Zhang et al., 2015; Jiao et al., 2016)	Homology (shared eukaryotic ancestor) with neo-functionalization in plants
RB/RBR	RB controls G1/S and G2/M checkpoints via E2F/DP; recruits PRC2 to deposit H3K27me3 at p16 locus; regulates proliferation and differentiation in stem cells (Kotake et al., 2007; Sage, 2012)	RBR controls cell cycle checkpoints via E2F/DP; directly represses WUS transcription; maintains stem cell niche in root and shoot meristems (Wildwater et al., 2005; Zhao et al., 2017; Desvoyes and Gutierrez, 2020)	Homology (deeply conserved eukaryotic cell-cycle regulator) with shared regulatory principle (targeting stem cell regulators)
Piwi/ZLL	Piwi maintains germline stem cell self-renewal; silences TEs via piRNAs; activates glycolytic enzyme mRNAs; modulates PRC2 binding (Cox et al., 1998; Rojas-Ríos et al., 2024; Claro-Linares and Rojas-Ríos, 2025)	ZLL maintains stem cell undifferentiated state; regulates STM spatial expression; potentiates WUS signaling (Moussian et al., 1998; Tucker et al., 2008)	Homology (Argonaute family) with functional analogy in stem cell maintenance
PcG/TrxG	PcG (PRC2/PRC1) silences lineage-specific genes in ESCs via H3K27me3; TrxG counteracts PcG to activate developmental genes (Schuettengruber et al., 2017)	PcG represses AGAMOUS, STM, and WUS via H3K27me3; TrxG (e.g., SYD) activates WUS transcription; SWI/SNF remodelers regulate meristem activity (Schubert et al., 2006; Kwon et al., 2005; Li et al., 2015)	Homology (evolutionarily conserved chromatin regulators) with shared regulatory principle (antagonistic control of stem cell genes)
SWI/SNF (BRM/SYD)	SWI/SNF complexes remodel chromatin; BRM (a TrxG component) antagonizes PcG repression; required for muscle stem cell function in mice (Albini et al., 2015; Kadoch et al., 2017)	BRM and SYD are ATPase subunits of SWI/SNF; SYD directly binds WUS promoter to activate transcription; BRM mutations impair root meristem activity (Kwon et al., 2005; Li et al., 2015; Wrona et al., 2024)	Homology with shared regulatory principle (chromatin remodeling for stem cell gene activation)
c-Myc/MYC	c-Myc cooperates with Oct4, Sox2, and Klf4 to reprogram somatic cells to iPSCs; regulates neural, hematopoietic, and cardiac stem cells (Yu et al., 2007; Conway et al., 2010; Curtis et al., 2012)	MYC (bHLH family) is negatively regulated by WUS; regulates meristematic stem cells; GhMYC3 orchestrates somatic embryogenesis via transcriptional cascade (Yang et al., 2012; Schuster et al., 2014; Yuan et al., 2023)	Homology (bHLH family) with shared regulatory principle (growth-promoting transcription factor) but divergent network context (regenerative vs. oncogenic)
Lin28/PpCSP1	Lin28, with Oct4/Sox2/Nanog, reprograms human fibroblasts to iPSCs; RNA-binding protein regulating mRNA metabolism (Yu et al., 2007; Tanabe et al., 2013)	PpCSP1 reprograms differentiated leaf cells to shoot apical stem cells; induced at wound sites; loss-of-function delays apical development (Li C. et al., 2017; Ishikawa et al., 2019; Ishikawa and Hasebe, 2022)	Functional analogy and sequence homology (RNA-binding proteins) with shared regulatory principle (reprogramming)
Pumilio (Pum)	Pum proteins regulate mRNA decay and translation via Pum recognition elements; maintain germline stem cells in *Drosophila*; double mutants impair ESC self-renewal in mice (Arvola et al., 2017; Uyhazi et al., 2020; Goldstrohm et al., 2018)	Pum homologs contribute to shoot apical stem cell maintenance in Arabidopsis (Francischini and Quaggio, 2009)	Homology (conserved RNA-binding domain) with shared regulatory principle (post-transcriptional control of stem cell fate)
Threonine metabolism	Threonine deprivation blocks mESC proliferation and drives differentiation; threonine catabolism supports 1C metabolism and H3K4 methylation (Wang et al., 2009; Ryu and Han, 2011; Sahoo et al., 2021)	Threonine is critical for root stem cell niche maintenance; THREONINE SYNTHASE1 mutants show reduced meristem activity (Reyes-Hernández et al., 2019)	Convergence (independent co-option of metabolic pathway for stem cell maintenance)
TOR/mTOR	mTOR integrates nutrients, energy, and hormones; activated by Akt; coordinates cell growth and cycle progression in stem cells (Fingar and Blenis, 2004; Limon and Fruman, 2012)	TOR regulates root/shoot growth and callus formation; activates WUS in a light-dependent manner; phosphorylates S6K2, which interacts with BIN2 (Pfeiffer et al., 2016; Li X. et al., 2017; Xiong et al., 2017)	Homology (conserved kinase) with shared regulatory principle (nutrient/energy sensing for stem cell activation)
GSK3β/BIN2	GSK3β phosphorylates β-catenin, marking it for degradation; inhibits stem cell renewal; GSK3β inhibitors promote chemical reprogramming (Latres et al., 1999; Hermida et al., 2017; Zhu et al., 2010)	BIN2 is the functional homolog of GSK3β; its inhibition promotes somatic embryogenesis in crop and forest species (Berenguer et al., 2021); BIN2 is phosphorylated by S6K2 downstream of TOR (Xiong et al., 2017)	Homology with shared regulatory principle (inhibition promotes stem cell reprogramming)
Nitric oxide (NO)	NO at low concentrations delays ESC differentiation and promotes survival; regulates differentiation via cGMP-dependent/independent pathways; critical for embryogenesis (Tejedo et al., 2010; Mujoo et al., 2011; Tranguch et al., 2003)	NO regulates root meristem growth and embryogenesis; represses WUS by disrupting AGO4-WUS interaction, fine-tuning SAM homeostasis (Sanz et al., 2014; Otvös et al., 2005; Zeng et al., 2023)	Convergence (independent use of NO as a concentration-dependent signaling molecule for stem cell control)

Abbreviations: BARD1, BRCA1-associated RING domain 1; AtROW1, BARD1 plant homolog; BRCA1, breast cancer gene 1; ESC, embryonic stem cell; WUS, WUSCHEL; WOX5, WUSCHEL-RELATED HOMEOBOX 5; H3K4me3, trimethylated histone H3 lysine 4; PRC2, Polycomb repressive complex 2; H3K27me3, trimethylated histone H3 lysine 27; RB, retinoblastoma protein; RBR, RETINOBLASTOMA-RELATED; E2F, eukaryotic 2 transcription factor; DP, dimerization partner; Piwi, P-element induced wimpy testis; ZLL, ZWILLE; TE, transposable element; piRNA, Piwi-interacting RNA; STM, SHOOT MERISTEMLESS; PcG, Polycomb group; TrxG, Trithorax group; SWI/SNF, switch/sucrose non-fermentable; BRM, BRAHMA; SYD, SPLAYD; ATPase, adenosine triphosphatase; c-Myc, MYC protooncogene; MYC, c-Myc plant homolog; bHLH, basic helix-loop-helix; iPSC, induced pluripotent stem cell; GhMYC3, Gossypium hirsutum MYC3; PpCSP1, cold-shock domain protein 1 of Physcomitrella patens; Pum, Pumilio; mESC, mouse embryonic stem cell; 1C, one-carbon; TOR, target of rapamycin; mTOR, mechanistic TOR; Akt, protein kinase B; S6K2, ribosomal protein S6 kinase β2; BIN2, BRASSINOSTEROID-INSENSITIVE 2; GSK3β, glycogen synthase kinase 3β; NO, nitric oxide; cGMP, cyclic guanosine monophosphate; AGO4, ARGONAUTE 4; SAM, shoot apical meristem.

Several lines of evidence support this perspective:

Evolutionary foundation: WUS and STM belong to the homeodomain family, a DNA-binding motif that is deeply conserved across plants and animals and constitutes a cornerstone of developmental patterning.

Metabolic-epigenetic interface: Threonine metabolism is vital for stem cells in both kingdoms and modulates the trimethylation of H3K4 (H3K4me3). In plants, the expression of WOX5, a root-specific WUS homolog, is regulated by H3K4me3. This implies that conserved metabolic pathways may influence stem cell regulators through analogous epigenetic mechanisms.

Integrated signaling hub: The conserved TOR kinase pathway is modulated by threonine through c-Myc in animals. Intriguingly, in plants, the c-Myc homolog (a bHLH transcription factor) is negatively regulated by WUS. Moreover, TOR kinase itself integrates light and metabolic signals to induce WUS overexpression, thereby stimulating stem cell proliferation and development. Consequently, WUS appears to be positioned as a central node within a conserved network linking metabolism and growth regulation.

Post-transcriptional and metabolic signaling interplay: NO fine-tunes stem cell activity at specific concentrations in both plants and animals. In *Arabidopsis*, NO mediates this effect partly by disrupting the AGO4–WUS interaction, which consequently represses WUS expression. Conversely, the argonaute family protein ZLL reinforces WUS signaling to maintain ESCs. Notably, the Piwi protein is essential for stem cell self-renewal in animals. Its structurally related homolog ZLL plays a pivotal role in stem cell homeostasis. While ZLL belongs to the same Argonaute protein family, its mechanistic action in plants may not fully recapitulate the piRNA-dependent pathways seen in animals.

Genomic integrity and cell cycle control: The BARD1/AtROW1 complex participates in DNA damage repair in animals and transcriptional repression in plants, thus directly targeting WUS for repression. Similarly, RBR binds to the *WUS* promoter during processes, such as meiotic differentiation, to prevent its ectopic expression. This highlights the control of stem cell fate via conserved cell cycle checkpoints.

Chromatin remodeling nexus: The antagonistic PcG and TrxG complexes, which regulate HOX genes in animals, also target WUS and STM in plants. PRC2 deposits H3K27me3 at the STM locus to define floral organ identity. Conversely, loss of the PRC2 catalytic subunits CLF or SWN results in ectopic silencing of WUS and collapse of the SAM. Furthermore, the TrxG-associated SWI/SNF chromatin remodeler can be recruited by c-Myc, which is regulated by WUS, thus creating a multi-layered feedback circuit. Additionally, the SWI/SNF-family ATPase SYD is directly recruited to the *WUS* promoter, where it energizes chromatin remodeling to activate WUS transcription.

Beyond the integration of WUS and STM, the contrasting network architectures of MYC homologs across kingdoms offer an additional example of how conserved molecular modules are repurposed for distinct biological outcomes. A recent study on somatic embryogenesis (SE) in cotton (Gossypium hirsutum) identified the bHLH transcription factor GhMYC3, a homolog of animal c-Myc, as a central pro-regenerative regulator (Yuan et al., 2023). Its activity is tightly controlled by the upstream antagonist GhRCD1, which binds and represses GhMYC3 to prevent its precocious activation. Upon relief of this inhibition, GhMYC3 operates within a defined transcriptional cascade to orchestrate orderly cell reprogramming and embryo formation. This multi-tiered regulatory architecture ensures that the conserved growth-promoting function of MYC is channeled toward controlled regenerative outcomes. In contrast, animal c-Myc is frequently subject to unchecked, upstream-driven activation in cancers, leading to uncontrolled proliferation and malignancy. Thus, the same core molecular function—MYC-mediated transcriptional regulation—can yield either regeneration or oncogenesis depending on its network context. This observation highlights a critical conceptual point: divergent biological outcomes arise not merely from the presence or absence of a given factor, but from how its activity is embedded within broader regulatory networks.

Notably, the translational relevance of such plant-derived regulatory principles is increasingly recognized. For instance, a plant immune protein was recently shown to enable broad antitumor responses in animals by rescuing microRNA deficiency, demonstrating that plant regulatory modules can be directly leveraged for cancer therapy (Qi et al., 2022). Collectively, these findings suggest that plant regulatory networks, including nodes such as the GhRCD1–GhMYC3 module, offer conceptually valuable frameworks for generating hypotheses that could be tested in animal systems. Whether such principles can eventually be translated into therapeutic strategies remains an open question and an exciting avenue for future investigation.

The recurrent integration of WUS/STM across these diverse pathways highlights a highly analogous ‘regulatory logic’ compared to animal stem cell networks. This does not necessarily imply that WUS/STM is the evolutionary ancestor of animal regulators, but rather suggests that both kingdoms have independently converged upon similar solutions—i.e., using deeply conserved transcriptional tools (like Homeodomains) and metabolic sensors (like TOR) to build kingdom-specific stem cell niches. The embedding of WUS/STM in conservative networks may provide a “regulatory logic” reference for animal research, rather than a direct molecular mechanism. These discoveries of parallel and intersecting regulatory relationships suggest that the core principles of stem cell regulation most likely evolved from a shared ancestral eukaryotic toolkit.

While our review highlights compelling parallels, it is equally important to acknowledge that the evidence for these convergences is not uniformly robust. The most direct and mechanistic evidence for shared stem cell regulators comes from the functional characterization of conserved protein families like RBR/RB and the PcG/TrxG systems. However, for other apparent convergences, such as the role of threonine metabolism or nitric oxide signaling, the supporting evidence is often indirect, derived from a limited number of model systems, and sometimes reliant on pharmacological inhibitors that may have off-target effects.

## 6 Conclusion and future directions

Based on the comparative analysis presented in this review, several promising directions for future research emerge. First, these findings contribute to an evolutionary framework for deciphering the origins and diversification of stem cell regulatory networks. Second, this work proposes a heuristic conceptual framework in which regulatory principles derived from plant stem cell biology could serve as a source of testable hypotheses for investigating animal stem cell behavior. This remains a speculative but potentially fruitful direction that merits further experimental exploration. Accordingly, future studies could investigate whether plant-specific metabolites or small RNAs that target conserved pathways modulate the behavior of mammalian stem cells. Moreover, investigating such cross-kingdom effect may yield novel tools for the precise manipulation of stem cell behavior.

In conclusion, this review highlights the profound evolutionary conservation that underpins stem cell biology across kingdoms. By elucidating these shared mechanisms, this review not only enhances the fundamental understanding of stem cell regulation but also fosters innovative interdisciplinary approaches that may accelerate progress in regenerative medicine and biotechnology.

## Glossary

BRCA1: breast cancer gene 1
RING: really interesting new gene
BARD1: BRCA1-associated RING Domain 1
AtROW1: BARD1 plant homolog
RB: retinoblastoma protein
RBR: RETINOBLASTOMA-RELATED (RB plant homolog)
Piwi: P-element induced wimpy testis
ZLL: ZWILLE (Piwi plant homolog)
GSK3β: glycogen synthase kinase
BIN2: BRASSINOSTEROID-INSENSITIVE 2 (GSK3β plant homolog)
c-Myc: MYC protooncogene
MYC: c-Myc plant homolog
WUS: WUSCHEL
STM: SHOOT MERISTEMLESS
SAM: shoot apical meristem
CLV3: CLAVATA3
Wnt: wingless-related integration site
LRP: low-density lipoprotein receptor-related protein
TOR: target of rapamycin
mTOR: mechanistic TOR
AKT: protein kinase B
E2F: eukaryotic 2 transcription factor
PRC: Polycomb repressive complex
H3K27me3: trimethylated histone H3 lysine 27
HOX: homeobox
PcG: Polycomb group
TrxG: Trithorax group
SWI/SNF: switch/sucrose non-fermentable
ATPase: adenosine triphosphatase
BRM: BRAHMA
SYD: SPLAYD
bHLH: basic helix-loop-helix
CLF: CURLY LEAF
SWN: SWINGER
PpCSP1: cold-shock domain protein 1 of *Physcomitrella patens*
Pum: Pumilio
1C: one-carbon
H3K4: histone H3 lysine 4
S6K2: ribosomal protein S6 kinase β2
NO: nitric oxide
cGMP: cyclic guanosine monophosphate
AGO4: ARGONAUTE 4

## References

[B1] Abe H. Yamaguchi-Shinozaki K. Urao T. Iwasaki T. Hosokawa D. Shinozaki K. (1997). Role of arabidopsis MYC and MYB homologs in drought- and abscisic acid-regulated gene expression. Plant Cell 9, 1859–1868. 10.1105/tpc.9.10.1859 9368419 PMC157027

[B2] Aida M. Ishida T. Tasaka M. (1999). Shoot apical meristem and cotyledon formation during *Arabidopsis* embryogenesis: interaction among the *CUP-SHAPED COTYLEDON* and *SHOOT MERISTEMLESS* genes. Development 126, 1563–1570. 10.1242/dev.126.8.1563 10079219

[B3] Albini S. Coutinho Toto P. Dall’Agnese A. Malecova B. Cenciarelli C. Felsani A. (2015). Brahma is required for cell cycle arrest and late muscle gene expression during skeletal myogenesis. EMBO Rep. 16, 1037–1050. 10.15252/embr.201540159 26136374 PMC4552495

[B4] Andersen P. Uosaki H. Shenje L. T. Kwon C. (2012). Non-canonical notch signaling: emerging role and mechanism. Trends Cell Biol. 22, 257–265. 10.1016/j.tcb.2012.02.003 22397947 PMC3348455

[B5] Arvola R. M. Weidmann C. A. Tanaka Hall T. M. Goldstrohm A. C. (2017). Combinatorial control of messenger RNAs by Pumilio, nanos and brain tumor proteins. RNA Biol. 14, 1445–1456. 10.1080/15476286.2017.1306168 28318367 PMC5785226

[B6] Arvola R. M. Chang C.-T. Buytendorp J. P. Levdansky Y. Valkov E. Freddolino L. (2020). Unique repression domains of Pumilio utilize deadenylation and decapping factors to accelerate destruction of target mRNAs. Nucleic Acids Res. 48, 1843–1871. 10.1093/nar/gkz1187 31863588 PMC7038932

[B7] Atta R. Laurens L. Boucheron‐Dubuisson E. Guivarc’h A. Carnero E. Giraudat‐Pautot V. (2009). Pluripotency of arabidopsis xylem pericycle underlies shoot regeneration from root and hypocotyl explants grown *in vitro* . Plant J. 57, 626–644. 10.1111/j.1365-313X.2008.03715.x 18980654

[B8] Batista M. R. Diniz P. Murta D. Torres A. Lopes-da-Costa L. Silva E. (2021). Balanced notch-wnt signaling interplay is required for mouse embryo and fetal development. Reproduction 161, 385–398. 10.1530/REP-20-0435 33539320

[B9] Beligni M. V. Lamattina L. (2000). Nitric oxide stimulates seed germination and de-etiolation, and inhibits hypocotyl elongation, three light-inducible responses in plants. Planta 210, 215–221. 10.1007/PL00008128 10664127

[B10] Benfey P. N. (1999). Stem cells: a tale of two kingdoms. Curr. Biol. 9, R171–R172. 10.1016/s0960-9822(99)80105-5 10074441

[B11] Berenguer E. Carneros E. Pérez-Pérez Y. Gil C. Martínez A. Testillano P. S. (2021). Small molecule inhibitors of mammalian GSK-3β promote *in vitro* plant cell reprogramming and somatic embryogenesis in crop and forest species. J. Exp. Bot. 72, 7808–7825. 10.1093/jxb/erab365 34338766 PMC8664590

[B12] Birnbaum K. D. Sánchez Alvarado A. (2008). Slicing across kingdoms: regeneration in plants and animals. Cell 132, 697–710. 10.1016/j.cell.2008.01.040 18295584 PMC2692308

[B13] Bisht S. Nigam M. Kunjwal S. S. Sergey P. Mishra A. P. Sharifi-Rad J. (2022). Cancer stem cells: from an insight into the basics to recent advances and therapeutic targeting. Stem Cells Int. 2022, 1–28. 10.1155/2022/9653244 35800881 PMC9256444

[B14] Boiani M. Schöler H. R. (2005). Regulatory networks in embryo-derived pluripotent stem cells. Nat. Rev. Mol. Cell Biol. 6, 872–881. 10.1038/nrm1744 16227977

[B15] Bolduc N. Hake S. (2009). The maize transcription factor KNOTTED1 directly regulates the gibberellin catabolism gene ga2ox1. Plant Cell 21, 1647–1658. 10.1105/tpc.109.068221 19567707 PMC2714931

[B16] Borghi L. Gutzat R. Fütterer J. Laizet Y. Hennig L. Gruissem W. (2010). Arabidopsis RETINOBLASTOMA-RELATED is required for stem cell maintenance, cell differentiation, and lateral organ production. Plant Cell 22, 1792–1811. 10.1105/tpc.110.074591 20525851 PMC2910961

[B17] Brand U. Grünewald M. Hobe M. Simon R. (2002). Regulation of *CLV3* expression by two homeobox genes in arabidopsis. Plant Physiol. 129, 565–575. 10.1104/pp.001867 12068101 PMC161677

[B18] Bustillo-Avendaño E. Ibáñez S. Sanz O. Sousa Barros J. A. Gude I. Perianez-Rodriguez J. (2018). Regulation of Hormonal Control, Cell Reprogramming, and Patterning during *de novo* Root Organogenesis. Plant Physiol. 176, 1709–1727. 10.1104/pp.17.00980 29233938 PMC5813533

[B19] Caballano-Infantes E. Cahuana G. M. Bedoya F. J. Salguero-Aranda C. Tejedo J. R. (2022). The role of nitric oxide in stem cell biology. Antioxidants (Basel) 11, 497. 10.3390/antiox11030497 35326146 PMC8944807

[B20] Cao X. Du Q. Guo Y. Wang Y. Jiao Y. (2023). Condensation of STM is critical for shoot meristem maintenance and salt tolerance in arabidopsis. Mol. Plant 16, 1445–1459. 10.1016/j.molp.2023.09.005 37674313

[B21] Chen D. Molitor A. Liu C. Shen W.-H. (2010). The arabidopsis PRC1-like ring-finger proteins are necessary for repression of embryonic traits during vegetative growth. Cell Res. 20, 1332–1344. 10.1038/cr.2010.151 21060339

[B22] Claro-Linares F. Rojas-Ríos P. (2025). PIWI proteins and piRNAs: key regulators of stem cell biology. Front. Cell Dev. Biol. 13, 1540313. 10.3389/fcell.2025.1540313 39981094 PMC11839606

[B23] Conway S. J. Firulli B. Firulli A. B. (2010). A bHLH code for cardiac morphogenesis. Pediatr. Cardiol. 31, 318–324. 10.1007/s00246-009-9608-x 20033146 PMC2837108

[B24] Costa S. Shaw P. (2007). “Open minded” cells: how cells can change fate. Trends Cell Biol. 17, 101–106. 10.1016/j.tcb.2006.12.005 17194589

[B25] Cox D. N. Chao A. Baker J. Chang L. Qiao D. Lin H. (1998). A novel class of evolutionarily conserved genes defined by piwi are essential for stem cell self-renewal. Genes Dev. 12, 3715–3727. 10.1101/gad.12.23.3715 9851978 PMC317255

[B26] Cui G. Li Y. Zheng L. Smith C. Bevan M. W. Li Y. (2024). The peptidase DA1 cleaves and destabilizes WUSCHEL to control shoot apical meristem size. Nat. Commun. 15, 4627. 10.1038/s41467-024-48361-7 38821962 PMC11143343

[B27] Curtis D. J. Salmon J. M. Pimanda J. E. (2012). Concise review: blood relatives: formation and regulation of hematopoietic stem cells by the basic helix-loop-helix transcription factors stem cell leukemia and lymphoblastic leukemia-derived sequence 1. Stem Cells 30, 1053–1058. 10.1002/stem.1093 22593015

[B28] De Mendoza A. Sebé-Pedrós A. (2019). Origin and evolution of eukaryotic transcription factors. Curr. Opin. Genet. and Dev. 58 (59), 25–32. 10.1016/j.gde.2019.07.010 31466037

[B29] Dello Ioio R. Nakamura K. Moubayidin L. Perilli S. Taniguchi M. Morita M. T. (2008). A genetic framework for the control of cell division and differentiation in the root meristem. Science 322, 1380–1384. 10.1126/science.1164147 19039136

[B30] Desvoyes B. Gutierrez C. (2020). Roles of plant retinoblastoma protein: cell cycle and beyond. EMBO J. 39, e105802. 10.15252/embj.2020105802 32865261 PMC7527812

[B31] El-Showk S. Ruonala R. Helariutta Y. (2013). Crossing paths: cytokinin signalling and crosstalk. Development 140, 1373–1383. 10.1242/dev.086371 23482484

[B32] Elledge S. J. Amon A. (2002). The BRCA1 suppressor hypothesis: an explanation for the tissue-specific tumor development in BRCA1 patients. Cancer Cell 1, 129–132. 10.1016/S1535-6108(02)00041-7 12086871

[B33] Fancy N. N. Bahlmann A. Loake G. J. (2017). Nitric oxide function in plant abiotic stress. Plant Cell and Environ. 40, 462–472. 10.1111/pce.12707 26754426

[B34] Fingar D. C. Blenis J. (2004). Target of rapamycin (TOR): an integrator of nutrient and growth factor signals and coordinator of cell growth and cell cycle progression. Oncogene 23, 3151–3171. 10.1038/sj.onc.1207542 15094765

[B35] Francischini C. W. Quaggio R. B. (2009). Molecular characterization of *Arabidopsis thaliana* PUF proteins--binding specificity and target candidates. FEBS J. 276, 5456–5470. 10.1111/j.1742-4658.2009.07230.x 19682068

[B36] Furutani M. Vernoux T. Traas J. Kato T. Tasaka M. Aida M. (2004). *PIN-FORMED1* and *PINOID* regulate boundary formation and cotyledon development in *Arabidopsis* embryogenesis. Development 131, 5021–5030. 10.1242/dev.01388 15371311

[B37] Galinha C. Hofhuis H. Luijten M. Willemsen V. Blilou I. Heidstra R. (2007). PLETHORA proteins as dose-dependent master regulators of Arabidopsis root development. Nature 449, 1053–1057. 10.1038/nature06206 17960244

[B38] Gallois J.-L. Woodward C. Reddy G. V. Sablowski R. (2002). Combined SHOOT MERISTEMLESS and WUSCHEL trigger ectopic organogenesis in *Arabidopsis* . Development 129, 3207–3217. 10.1242/dev.129.13.3207 12070095

[B39] Goldstrohm A. C. Hall T. M. T. McKenney K. M. (2018). Post-transcriptional regulatory functions of Mammalian pumilio proteins. Trends Genet. 34, 972–990. 10.1016/j.tig.2018.09.006 30316580 PMC6251728

[B40] Gordon S. P. Heisler M. G. Reddy G. V. Ohno C. Das P. Meyerowitz E. M. (2007). Pattern formation during *de novo* assembly of the *Arabidopsis* shoot meristem. Development 134, 3539–3548. 10.1242/dev.010298 17827180

[B41] Gordon S. P. Chickarmane V. S. Ohno C. Meyerowitz E. M. (2009). Multiple feedback loops through cytokinin signaling control stem cell number within the *Arabidopsis* shoot meristem. Proc. Natl. Acad. Sci. U.S.A. 106, 16529–16534. 10.1073/pnas.0908122106 19717465 PMC2752578

[B42] Greb T. Lohmann J. U. (2016). Plant stem cells. Curr. Biol. 26, R816–R821. 10.1016/j.cub.2016.07.070 27623267

[B43] Gutzat R. Borghi L. Fütterer J. Bischof S. Laizet Y. Hennig L. (2011). RETINOBLASTOMA-RELATED PROTEIN controls the transition to autotrophic plant development. Development 138, 2977–2986. 10.1242/dev.060830 21693514

[B44] Gutzat R. Borghi L. Gruissem W. (2012). Emerging roles of RETINOBLASTOMA-RELATED proteins in evolution and plant development. Trends Plant Sci. 17, 139–148. 10.1016/j.tplants.2011.12.001 22240181

[B45] Han P. Li Q. Zhu Y.-X. (2008). Mutation of arabidopsis BARD1 causes meristem defects by failing to confine WUSCHEL expression to the organizing center. Plant Cell 20, 1482–1493. 10.1105/tpc.108.058867 18591352 PMC2483370

[B46] Haugen R. J. Barnier C. Elrod N. D. Luo H. Jensen M. K. Ji P. (2024). Regulation of the drosophila transcriptome by Pumilio and the CCR4-NOT deadenylase complex. RNA 30, 866–890. 10.1261/rna.079813.123 38627019 PMC11182014

[B47] He Y. Tang R.-H. Hao Y. Stevens R. D. Cook C. W. Ahn S. M. (2004). Nitric oxide represses the *arabidopsis* floral transition. Science 305, 1968–1971. 10.1126/science.1098837 15448272

[B48] Heisler M. G. Ohno C. Das P. Sieber P. Reddy G. V. Long J. A. (2005). Patterns of auxin transport and gene expression during primordium development revealed by live imaging of the Arabidopsis inflorescence meristem. Curr. Biol. 15, 1899–1911. 10.1016/j.cub.2005.09.052 16271866

[B49] Hermida M. A. Dinesh Kumar J. Leslie N. R. (2017). GSK3 and its interactions with the PI3K/AKT/mTOR signalling network. Adv. Biol. Regul. 65, 5–15. 10.1016/j.jbior.2017.06.003 28712664

[B50] Ikeuchi M. Ogawa Y. Iwase A. Sugimoto K. (2016). Plant regeneration: cellular origins and molecular mechanisms. Development 143, 1442–1451. 10.1242/dev.134668 27143753

[B51] Ishikawa M. Hasebe M. (2022). Molecular mechanisms of reprogramming of differentiated cells into stem cells in the moss Physcomitrium patens. Curr. Opin. Plant Biol. 65, 102123. 10.1016/j.pbi.2021.102123 34735974

[B52] Ishikawa M. Morishita M. Higuchi Y. Ichikawa S. Ishikawa T. Nishiyama T. (2019). Physcomitrella STEMIN transcription factor induces stem cell formation with epigenetic reprogramming. Nat. Plants 5, 681–690. 10.1038/s41477-019-0464-2 31285563

[B53] Jasinski S. Piazza P. Craft J. Hay A. Woolley L. Rieu I. (2005). KNOX action in Arabidopsis is mediated by coordinate regulation of cytokinin and gibberellin activities. Curr. Biol. 15, 1560–1565. 10.1016/j.cub.2005.07.023 16139211

[B54] Jiao Y. Zhang Y. Zhu Y.-X. (2016). Recent advances in the research for the homolog of breast cancer associated gene AtROW1 in higher plants. Sci. China Life Sci. 59, 825–831. 10.1007/s11427-016-5086-6 27502904

[B55] Kadoch C. Williams R. T. Calarco J. P. Miller E. L. Weber C. M. Braun S. M. G. (2017). Dynamics of BAF-polycomb complex opposition on heterochromatin in normal and oncogenic states. Nat. Genet. 49, 213–222. 10.1038/ng.3734 27941796 PMC5285326

[B56] Katoh M. (2017). Canonical and non-canonical WNT signaling in cancer stem cells and their niches: cellular heterogeneity, omics reprogramming, targeted therapy and tumor plasticity. Int. J. Oncol. 51, 1357–1369. (Review). 10.3892/ijo.2017.4129 29048660 PMC5642388

[B57] Katoh M. Katoh M. (2006). Notch ligand, JAG1, is evolutionarily conserved target of canonical WNT signaling pathway in progenitor cells. Int. J. Mol. Med. 17, 681–685. 10.3892/ijmm.17.4.681 16525728

[B58] Kotake Y. Cao R. Viatour P. Sage J. Zhang Y. Xiong Y. (2007). pRB family proteins are required for H3K27 trimethylation and polycomb repression complexes binding to and silencing p16 ^INK4a^ tumor suppressor gene. Genes Dev. 21, 49–54. 10.1101/gad.1499407 17210787 PMC1759899

[B59] Kwon C. S. Chen C. Wagner D. (2005). WUSCHEL is a primary target for transcriptional regulation by SPLAYED in dynamic control of stem cell fate in arabidopsis. Genes Dev. 19, 992–1003. 10.1101/gad.1276305 15833920 PMC1080137

[B60] Kwon C. Cheng P. King I. N. Andersen P. Shenje L. Nigam V. (2011). Notch post-translationally regulates β-catenin protein in stem and progenitor cells. Nat. Cell Biol. 13, 1244–1251. 10.1038/ncb2313 21841793 PMC3187850

[B61] Lai H. C. Meredith D. M. Johnson J. E. (2013). “bHLH factors in neurogenesis and neuronal subtype specification,” in Patterning and Cell Type Specification in the Developing CNS and PNS (Elsevier), 333–354. 10.1016/B978-0-12-397265-1.00065-4

[B62] Lampreia F. P. Carmelo J. G. Anjos-Afonso F. (2017). Notch signaling in the regulation of hematopoietic stem cell. Curr. Stem Cell Rep. 3, 202–209. 10.1007/s40778-017-0090-8 28845387 PMC5548842

[B63] Latres E. Chiaur D. S. Pagano M. (1999). The human F box protein β-Trcp associates with the Cul1/Skp1 complex and regulates the stability of β-catenin. Oncogene 18, 849–854. 10.1038/sj.onc.1202653 10023660

[B64] Laux T. Mayer K. F. Berger J. Jürgens G. (1996). The WUSCHEL gene is required for shoot and floral meristem integrity in arabidopsis. Development 122, 87–96. 10.1242/dev.122.1.87 8565856

[B65] Lee K. Seo P. J. (2017). Arabidopsis TOR signaling is essential for sugar-regulated callus formation. J. Integr. Plant Biol. 59, 742–746. 10.1111/jipb.12560 28623850

[B66] Lee K.-S. Wu Z. Song Y. Mitra S. S. Feroze A. H. Cheshier S. H. (2013). Roles of PINK1, mTORC2, and mitochondria in preserving brain tumor-forming stem cells in a noncanonical notch signaling pathway. Genes Dev. 27, 2642–2647. 10.1101/gad.225169.113 24352421 PMC3877754

[B67] Lenhard M. Jürgens G. Laux T. (2002). The *WUSCHEL* and *SHOOTMERISTEMLESS* genes fulfil complementary roles in *arabidopsis* shoot meristem regulation. Development 129, 3195–3206. 10.1242/dev.129.13.3195 12070094

[B68] Li C. Chen C. Gao L. Yang S. Nguyen V. Shi X. (2015). The arabidopsis SWI2/SNF2 chromatin remodeler BRAHMA regulates polycomb function during vegetative development and directly activates the flowering repressor gene SVP. PLoS Genet. 11, e1004944. 10.1371/journal.pgen.1004944 25615622 PMC4304717

[B69] Li C. Sako Y. Imai A. Nishiyama T. Thompson K. Kubo M. (2017). A Lin28 homologue reprograms differentiated cells to stem cells in the moss physcomitrella patens. Nat. Commun. 8, 14242. 10.1038/ncomms14242 28128346 PMC5290140

[B70] Li X. Cai W. Liu Y. Li H. Fu L. Liu Z. (2017). Differential TOR activation and cell proliferation in arabidopsis root and shoot apexes. Proc. Natl. Acad. Sci. U. S. A. 114, 2765–2770. 10.1073/pnas.1618782114 28223530 PMC5347562

[B71] Li P. Song L. Ma L. Han C. Li L. Lu L.-Y. (2025). BRCA1 preserves genome integrity during the formation of undifferentiated spermatogonia. EMBO Rep. 26, 3747–3772. 10.1038/s44319-025-00487-5 40437289 PMC12332178

[B72] Li-Bao L. Díaz-Díaz C. Raiola M. Sierra R. Temiño S. Moya F. J. (2024). Regulation of Myc transcription by an enhancer cluster dedicated to pluripotency and early embryonic expression. Nat. Commun. 15, 3931. 10.1038/s41467-024-48258-5 38729993 PMC11087473

[B73] Limon J. J. Fruman D. A. (2012). Akt and mTOR in B cell activation and differentiation. Front. Immunol. 3, 228. 10.3389/fimmu.2012.00228 22888331 PMC3412259

[B74] Lin X. Yuan C. Zhu B. Yuan T. Li X. Yuan S. (2021). LFR physically and genetically interacts with SWI/SNF component SWI3B to regulate leaf blade development in arabidopsis. Front. Plant Sci. 12, 717649. 10.3389/fpls.2021.717649 34456957 PMC8385146

[B75] Lin X. Yuan T. Guo H. Guo Y. Yamaguchi N. Wang S. (2023). The regulation of chromatin configuration at AGAMOUS locus by LFR-SYD-containing complex is critical for reproductive organ development in arabidopsis. Plant J. 116, 478–496. 10.1111/tpj.16385 37478313

[B76] Mähönen A. P. Ten Tusscher K. Siligato R. Smetana O. Díaz-Triviño S. Salojärvi J. (2014). PLETHORA gradient formation mechanism separates auxin responses. Nature 515, 125–129. 10.1038/nature13663 25156253 PMC4326657

[B77] Makkena S. Lamb R. S. (2013). The bHLH transcription factor SPATULA regulates root growth by controlling the size of the root meristem. BMC Plant Biol. 13, 1. 10.1186/1471-2229-13-1 23280064 PMC3583232

[B78] Maren N. A. Duan H. Da K. Yencho G. C. Ranney T. G. Liu W. (2022). Genotype-independent plant transformation. Hortic. Res. 9, uhac047. 10.1093/hr/uhac047 35531314 PMC9070643

[B79] Marletta M. A. (1994). Nitric oxide synthase: aspects concerning structure and catalysis. Cell 78, 927–930. 10.1016/0092-8674(94)90268-2 7522970

[B80] Matos J. L. Bergmann D. C. (2014). Convergence of stem cell behaviors and genetic regulation between animals and plants: insights from the *Arabidopsis thaliana* stomatal lineage. F1000Prime Rep. 6, 53. 10.12703/P6-53 25184043 PMC4108953

[B81] Metcalfe C. Mendoza-Topaz C. Mieszczanek J. Bienz M. (2010). Stability elements in the LRP6 cytoplasmic tail confer efficient signalling upon DIX-dependent polymerization. J. Cell Sci. 123, 1588–1599. 10.1242/jcs.067546 20388731 PMC2858023

[B82] Meyerowitz E. M. (2002). Plants compared to animals: the broadest comparative study of development. Science 295, 1482–1485. 10.1126/science.1066609 11859185

[B83] Mills K. M. Szczerkowski J. L. A. Habib S. J. (2017). Wnt ligand presentation and reception: from the stem cell niche to tissue engineering. Open Biol. 7, 170140. 10.1098/rsob.170140 28814649 PMC5577451

[B84] Moubayidin L. Di Mambro R. Sabatini S. (2009). Cytokinin–auxin crosstalk. Trends Plant Sci. 14, 557–562. 10.1016/j.tplants.2009.06.010 19734082

[B85] Moussian B. Schoof H. Haecker A. Jürgens G. Laux T. (1998). Role of the ZWILLE gene in the regulation of central shoot meristem cell fate during arabidopsis embryogenesis. EMBO J. 17, 1799–1809. 10.1093/emboj/17.6.1799 9501101 PMC1170527

[B86] Muchardt C. Yaniv M. (1999). ATP-dependent chromatin remodelling: SWI/SNF and co. are on the job. J. Mol. Biol. 293, 187–198. 10.1006/jmbi.1999.2999 10529347

[B87] Mujoo K. Krumenacker J. S. Murad F. (2011). Nitric oxide-cyclic GMP signaling in stem cell differentiation. Free Radic. Biol. Med. 51, 2150–2157. 10.1016/j.freeradbiomed.2011.09.037 22019632 PMC3232180

[B88] Niedzialkowska E. Liu L. Kuscu C. Mayo Z. Minor W. Strahl B. D. (2022). Tip60 acetylation of histone H3K4 temporally controls chromosome passenger complex localization. Mol. Biol. Cell 33, br15. 10.1091/mbc.E21-06-0283 35653296 PMC9582641

[B89] Olariu V. Nilsson J. Jönsson H. Peterson C. (2017). Different reprogramming propensities in plants and mammals: are small variations in the core network wirings responsible? PLoS ONE 12, e0175251. 10.1371/journal.pone.0175251 28384293 PMC5383272

[B90] Otte A. P. Kwaks T. H. J. (2003). Gene repression by polycomb group protein complexes: a distinct complex for every occasion? Curr. Opin. Genet. Dev. 13, 448–454. 10.1016/s0959-437x(03)00108-4 14550408

[B91] Otvös K. Pasternak T. P. Miskolczi P. Domoki M. Dorjgotov D. Szucs A. (2005). Nitric oxide is required for, and promotes auxin-mediated activation of, cell division and embryogenic cell formation but does not influence cell cycle progression in alfalfa cell cultures. Plant J. 43, 849–860. 10.1111/j.1365-313X.2005.02494.x 16146524

[B92] Perumalsamy L. R. Nagala M. Banerjee P. Sarin A. (2009). A hierarchical cascade activated by non-canonical notch signaling and the mTOR–Rictor complex regulates neglect-induced death in mammalian cells. Cell Death Differ. 16, 879–889. 10.1038/cdd.2009.20 19265851

[B93] Pfeiffer A. Janocha D. Dong Y. Medzihradszky A. Schöne S. Daum G. (2016). Integration of light and metabolic signals for stem cell activation at the shoot apical meristem. Elife 5, e17023. 10.7554/eLife.17023 27400267 PMC4969040

[B94] Phan N. Zaytseva Y. Lin C.-C. Mishra M. Sun W. Pawlica P. (2025). PUM2 binds SARS-CoV-2 RNA and PUM1 mildly reduces viral RNA levels, but neither protein affects progeny virus production. J. Gen. Virol. 106, 002152. 10.1099/jgv.0.002152 40956600 PMC12441121

[B95] Pi L. Aichinger E. van der Graaff E. Llavata-Peris C. I. Weijers D. Hennig L. (2015). Organizer-derived WOX5 signal maintains root columella stem cells through chromatin-mediated repression of CDF4 expression. Dev. Cell 33, 576–588. 10.1016/j.devcel.2015.04.024 26028217

[B96] Pien S. Grossniklaus U. (2007). Polycomb group and trithorax group proteins in arabidopsis. Biochim. Biophys. Acta 1769, 375–382. 10.1016/j.bbaexp.2007.01.010 17363079

[B97] Qi Y. Ding L. Zhang S. Yao S. Ong J. Li Y. (2022). A plant immune protein enables broad antitumor response by rescuing microRNA deficiency. Cell 185, 1888–1904.e24. 10.1016/j.cell.2022.04.030 35623329

[B98] Qin L. Yin Y.-T. Zheng F.-J. Peng L.-X. Yang C.-F. Bao Y.-N. (2015). WNT5A promotes stemness characteristics in nasopharyngeal carcinoma cells leading to metastasis and tumorigenesis. Oncotarget 6, 10239–10252. 10.18632/oncotarget.3518 25823923 PMC4496352

[B99] Raff M. (2003). Adult stem cell plasticity: fact or artifact? Annu. Rev. Cell Dev. Biol. 19, 1–22. 10.1146/annurev.cellbio.19.111301.143037 14570561

[B100] Reidt W. Wurz R. Wanieck K. Chu H. H. Puchta H. (2006). A homologue of the breast cancer-associated gene BARD1 is involved in DNA repair in plants. EMBO J. 25, 4326–4337. 10.1038/sj.emboj.7601313 16957774 PMC1570427

[B101] Reyes-Hernández B. J. Shishkova S. Amir R. Quintana-Armas A. X. Napsucialy-Mendivil S. Cervantes-Gamez R. G. (2019). Root stem cell niche maintenance and apical meristem activity critically depend on THREONINE SYNTHASE1. J. Exp. Bot. 70, 3835–3849. 10.1093/jxb/erz165 30972413

[B102] Rodríguez-Leal D. Castillo-Cobián A. Rodríguez-Arévalo I. Vielle-Calzada J.-P. (2016). A primary sequence analysis of the ARGONAUTE protein family in plants. Front. Plant Sci. 7, 1347. 10.3389/fpls.2016.01347 27635128 PMC5007885

[B103] Rojas-Ríos P. Simonelig M. (2018). piRNAs and PIWI proteins: regulators of gene expression in development and stem cells. Development 145, dev161786. 10.1242/dev.161786 30194260

[B104] Rojas-Ríos P. Chartier A. Enjolras C. Cremaschi J. Garret C. Boughlita A. (2024). piRNAs are regulators of metabolic reprogramming in stem cells. Nat. Commun. 15, 8405. 10.1038/s41467-024-52709-4 39333531 PMC11437085

[B105] Rosspopoff O. Chelysheva L. Saffar J. Lecorgne L. Gey D. Caillieux E. (2017). Direct conversion of root primordium into shoot meristem relies on timing of stem cell niche development. Development 144, 1187–1200. 10.1242/dev.142570 28174250

[B106] Ryu J. M. Han H. J. (2011). L-threonine regulates G1/S phase transition of mouse embryonic stem cells *via* PI3K/Akt, MAPKs, and mTORC pathways. J. Biol. Chem. 286, 23667–23678. 10.1074/jbc.M110.216283 21550972 PMC3129147

[B107] Sage J. (2012). The retinoblastoma tumor suppressor and stem cell biology. Genes Dev. 26, 1409–1420. 10.1101/gad.193730.112 22751497 PMC3403009

[B108] Sahoo D. P. Van Winkle L. J. Díaz De La Garza R. I. Dubrovsky J. G. (2021). Interkingdom comparison of threonine metabolism for stem cell maintenance in plants and animals. Front. Cell Dev. Biol. 9, 672545. 10.3389/fcell.2021.672545 34557481 PMC8454773

[B109] Sanz L. Fernández-Marcos M. Modrego A. Lewis D. R. Muday G. K. Pollmann S. (2014). Nitric oxide plays a role in stem cell niche homeostasis through its interaction with auxin. Plant Physiol. 166, 1972–1984. 10.1104/pp.114.247445 25315603 PMC4256006

[B110] Schneuwly S. Kuroiwa A. Baumgartner P. Gehring W. J. (1986). Structural organization and sequence of the homeotic gene antennapedia of *Drosophila melanogaster* . EMBO J. 5, 733–739. 10.1002/j.1460-2075.1986.tb04275.x 10408949 PMC1166852

[B111] Schubert D. Primavesi L. Bishopp A. Roberts G. Doonan J. Jenuwein T. (2006). Silencing by plant Polycomb-group genes requires dispersed trimethylation of histone H3 at lysine 27. EMBO J. 25, 4638–4649. 10.1038/sj.emboj.7601311 16957776 PMC1590001

[B112] Schuettengruber B. Bourbon H.-M. Di Croce L. Cavalli G. (2017). Genome regulation by polycomb and trithorax: 70 years and counting. Cell 171, 34–57. 10.1016/j.cell.2017.08.002 28938122

[B113] Schuster C. Gaillochet C. Medzihradszky A. Busch W. Daum G. Krebs M. (2014). A regulatory framework for shoot stem cell control integrating metabolic, transcriptional, and phytohormone signals. Dev. Cell 28, 438–449. 10.1016/j.devcel.2014.01.013 24576426

[B114] Shahin W. S. Ebed S. O. Tyler S. R. Miljkovic B. Choi S. H. Zhang Y. (2023). Redox-dependent Igfbp2 signaling controls Brca1 DNA damage response to govern neural stem cell fate. Nat. Commun. 14, 444. 10.1038/s41467-023-36174-z 36707536 PMC9883463

[B115] Shi Q. Xue C. Zeng Y. Yuan X. Chu Q. Jiang S. (2024). Notch signaling pathway in cancer: from mechanistic insights to targeted therapies. Sig Transduct. Target Ther. 9, 128. 10.1038/s41392-024-01828-x 38797752 PMC11128457

[B116] Shpak E. D. Uzair M. (2025). WUSCHEL: the essential regulator of the arabidopsis shoot apical meristem. Curr. Opin. Plant Biol. 85, 102739. 10.1016/j.pbi.2025.102739 40381531

[B117] Simon J. A. Tamkun J. W. (2002). Programming off and on states in chromatin: mechanisms of polycomb and trithorax group complexes. Curr. Opin. Genet. Dev. 12, 210–218. 10.1016/s0959-437x(02)00288-5 11893495

[B118] Spassov D. S. Jurecic R. (2003). Mouse Pum1 and Pum2 genes, members of the Pumilio family of RNA-binding proteins, show differential expression in fetal and adult hematopoietic stem cells and progenitors. Blood Cells Mol. Dis. 30, 55–69. 10.1016/s1079-9796(03)00003-2 12667987

[B119] Stanton B. Z. Hodges C. Calarco J. P. Braun S. M. G. Ku W. L. Kadoch C. (2017). Smarca4 ATPase mutations disrupt direct eviction of PRC1 from chromatin. Nat. Genet. 49, 282–288. 10.1038/ng.3735 27941795 PMC5373480

[B120] Stöhr C. G. Steffens S. Polifka I. Jung R. Kahlmeyer A. Ivanyi P. (2019). Piwi-like 1 protein expression is a prognostic factor for renal cell carcinoma patients. Sci. Rep. 9, 1741. 10.1038/s41598-018-38254-3 30741998 PMC6370845

[B121] Stolyarenko A. D. (2020). Nuclear argonaute piwi gene mutation affects rRNA by inducing rRNA fragment accumulation, antisense expression, and defective processing in drosophila ovaries. Int. J. Mol. Sci. 21, 1119. 10.3390/ijms21031119 32046213 PMC7037970

[B122] Su Y. H. Zhao X. Y. Liu Y. B. Zhang C. L. O’Neill S. D. Zhang X. S. (2009). Auxin‐induced *WUS* expression is essential for embryonic stem cell renewal during somatic embryogenesis in arabidopsis. Plant J. 59, 448–460. 10.1111/j.1365-313X.2009.03880.x 19453451 PMC2788036

[B123] Su Y. H. Zhou C. Li Y. J. Yu Y. Tang L. P. Zhang W. J. (2020). Integration of pluripotency pathways regulates stem cell maintenance in the *Arabidopsis* shoot meristem. Proc. Natl. Acad. Sci. U.S.A. 117, 22561–22571. 10.1073/pnas.2015248117 32839309 PMC7486707

[B124] Tanabe K. Nakamura M. Narita M. Takahashi K. Yamanaka S. (2013). Maturation, not initiation, is the major roadblock during reprogramming toward pluripotency from human fibroblasts. Proc. Natl. Acad. Sci. U. S. A. 110, 12172–12179. 10.1073/pnas.1310291110 23812749 PMC3725041

[B125] Tejedo J. R. Tapia-Limonchi R. Mora-Castilla S. Cahuana G. M. Hmadcha A. Martin F. (2010). Low concentrations of nitric oxide delay the differentiation of embryonic stem cells and promote their survival. Cell Death Dis. 1, e80. 10.1038/cddis.2010.57 21368853 PMC3035898

[B126] Tranguch S. Steuerwald N. Huet-Hudson Y. M. (2003). Nitric oxide synthase production and nitric oxide regulation of preimplantation embryo development. Biol. Reprod. 68, 1538–1544. 10.1095/biolreprod.102.009282 12606428

[B127] Tucker M. R. Hinze A. Tucker E. J. Takada S. Jürgens G. Laux T. (2008). Vascular signalling mediated by ZWILLE potentiates WUSCHEL function during shoot meristem stem cell development in the Arabidopsis embryo. Development 135, 2839–2843. 10.1242/dev.023648 18653559

[B128] Uyhazi K. E. Yang Y. Liu N. Qi H. Huang X. A. Mak W. (2020). Pumilio proteins utilize distinct regulatory mechanisms to achieve complementary functions required for pluripotency and embryogenesis. Proc. Natl. Acad. Sci. U. S. A. 117, 7851–7862. 10.1073/pnas.1916471117 32198202 PMC7148564

[B129] Van Camp J. K. Beckers S. Zegers D. Van Hul W. (2014). Wnt signaling and the control of human stem cell fate. Stem Cell Rev Rep 10, 207–229. 10.1007/s12015-013-9486-8 24323281

[B130] Wang J. Alexander P. Wu L. Hammer R. Cleaver O. McKnight S. L. (2009). Dependence of mouse embryonic stem cells on threonine catabolism. Science 325, 435–439. 10.1126/science.1173288 19589965 PMC4373593

[B131] Wang M.-T. Holderfield M. Galeas J. Delrosario R. To M. D. Balmain A. (2015). K-Ras promotes tumorigenicity through suppression of non-canonical wnt signaling. Cell 163, 1237–1251. 10.1016/j.cell.2015.10.041 26590425

[B132] Wang X. Lin D.-H. Yan Y. Wang A.-H. Liao J. Meng Q. (2023). The PIWI-specific insertion module helps load longer piRNAs for translational activation essential for male fertility. Sci. China Life Sci. 66, 1459–1481. 10.1007/s11427-023-2390-5 37335463

[B133] Wang J. Zhou P.-H. Li C.-H. Liang Y.-L. Liu G.-Z. Yang S.-C. (2024). Progress on medicinal plant regeneration and the road ahead. Med. Plant Biol. 3, 0. 10.48130/mpb-0024-0026

[B134] Weidmann C. A. Qiu C. Arvola R. M. Lou T.-F. Killingsworth J. Campbell Z. T. (2016). Drosophila nanos acts as a molecular clamp that modulates the RNA-binding and repression activities of Pumilio. Elife 5, e17096. 10.7554/eLife.17096 27482653 PMC4995099

[B135] Wildwater M. Campilho A. Perez-Perez J. M. Heidstra R. Blilou I. Korthout H. (2005). The retinoblastoma-related gene regulates stem cell maintenance in arabidopsis roots. Cell 123, 1337–1349. 10.1016/j.cell.2005.09.042 16377572

[B136] Wittmer J. Heidstra R. (2024). Appreciating animal induced pluripotent stem cells to shape plant cell reprogramming strategies. J. Exp. Bot. 75, 4373–4393. 10.1093/jxb/erae264 38869461 PMC11263491

[B137] Wrona M. Zinsmeister J. Krzyszton M. Villette C. Zumsteg J. Mercier P. (2024). The BRAHMA-associated SWI/SNF chromatin remodeling complex controls Arabidopsis seed quality and physiology. Plant Physiol. 197, kiae642. 10.1093/plphys/kiae642 39661382 PMC11668257

[B138] Xiong F. Zhang R. Meng Z. Deng K. Que Y. Zhuo F. (2017). Brassinosteriod insensitive 2 (BIN2) acts as a downstream effector of the target of rapamycin (TOR) signaling pathway to regulate photoautotrophic growth in arabidopsis. New Phytol. 213, 233–249. 10.1111/nph.14118 27479935

[B139] Xu F. Zhang D. Le L. Pu L. (2024). Polycomb and trithorax: their yin-yang dynamics in plants. Mol. Plant 17, 845–847. 10.1016/j.molp.2024.05.005 38783605

[B140] Yan T. Hou Q. Wei X. Qi Y. Pu A. Wu S. (2023). Promoting genotype-independent plant transformation by manipulating developmental regulatory genes and/or using nanoparticles. Plant Cell Rep. 42, 1395–1417. 10.1007/s00299-023-03037-2 37311877 PMC10447291

[B141] Yang F. Wang Q. Schmitz G. Müller D. Theres K. (2012). The bHLH protein ROX acts in concert with RAX1 and LAS to modulate axillary meristem formation in arabidopsis. Plant J. 71, 61–70. 10.1111/j.1365-313X.2012.04970.x 22372440

[B142] Yang L. Shi P. Zhao G. Xu J. Peng W. Zhang J. (2020). Targeting cancer stem cell pathways for cancer therapy. Sig Transduct. Target Ther. 5, 8. 10.1038/s41392-020-0110-5 32296030 PMC7005297

[B143] Ye W. Lin W. Tartakoff A. M. Tao T. (2011). Karyopherins in nuclear transport of homeodomain proteins during development. Biochim. Biophys. Acta 1813, 1654–1662. 10.1016/j.bbamcr.2011.01.013 21256166 PMC3628554

[B144] Yu J. Vodyanik M. A. Smuga-Otto K. Antosiewicz-Bourget J. Frane J. L. Tian S. (2007). Induced pluripotent stem cell lines derived from human somatic cells. Science 318, 1917–1920. 10.1126/science.1151526 18029452

[B145] Yu M. Lamattina L. Spoel S. H. Loake G. J. (2014). Nitric oxide function in plant biology: a redox cue in deconvolution. New Phytol. 202, 1142–1156. 10.1111/nph.12739 24611485

[B146] Yuan J. Liu X. Zhao H. Wang Y. Wei X. Wang P. (2023). GhRCD1 regulates cotton somatic embryogenesis by modulating the GhMYC3-GhMYB44-GhLBD18 transcriptional cascade. New Phytol. 240, 207–223. 10.1111/nph.19120 37434324

[B147] Zamora-Zaragoza J. Klap K. Sánchez-Pérez J. Vielle-Calzada J.-P. Willemsen V. Scheres B. (2024). Developmental cues are encoded by the combinatorial phosphorylation of arabidopsis RETINOBLASTOMA-RELATED protein RBR1. EMBO J. 43, 6656–6678. 10.1038/s44318-024-00282-3 39468281 PMC11649800

[B148] Zeng J. Zhao X. Liang Z. Hidalgo I. Gebert M. Fan P. (2023). Nitric oxide controls shoot meristem activity *via* regulation of DNA methylation. Nat. Commun. 14, 8001. 10.1038/s41467-023-43705-1 38049411 PMC10696095

[B149] Zhang Y. Jiao Y. Liu Z. Zhu Y.-X. (2015). ROW1 maintains quiescent centre identity by confining WOX5 expression to specific cells. Nat. Commun. 6, 6003. 10.1038/ncomms7003 25631790 PMC4316744

[B150] Zhang T.-Q. Lian H. Zhou C.-M. Xu L. Jiao Y. Wang J.-W. (2017). A Two-Step Model for *de novo* Activation of *WUSCHEL* during Plant Shoot Regeneration. Plant Cell 29, 1073–1087. 10.1105/tpc.16.00863 28389585 PMC5466026

[B151] Zhao Y. (2018). Essential roles of local auxin biosynthesis in plant development and in adaptation to environmental changes. Annu. Rev. Plant Biol. 69, 417–435. 10.1146/annurev-arplant-042817-040226 29489397

[B152] Zhao X. Bramsiepe J. Van Durme M. Komaki S. Prusicki M. A. Maruyama D. (2017). RETINOBLASTOMA RELATED1 mediates germline entry in arabidopsis. Science 356, eaaf6532. 10.1126/science.aaf6532 28450583

[B153] Zhou Y. Wang Y. Krause K. Yang T. Dongus J. A. Zhang Y. (2018). Telobox motifs recruit CLF/SWN–PRC2 for H3K27me3 deposition *via* TRB factors in arabidopsis. Nat. Genet. 50, 638–644. 10.1038/s41588-018-0109-9 29700471

[B154] Zhou Y. Tao L. Qiu J. Xu J. Yang X. Zhang Y. (2024). Tumor biomarkers for diagnosis, prognosis and targeted therapy. Sig Transduct. Target Ther. 9, 132. 10.1038/s41392-024-01823-2 PMC11102923 38763973

[B155] Zhu S. Li W. Zhou H. Wei W. Ambasudhan R. Lin T. (2010). Reprogramming of human primary somatic cells by OCT4 and chemical compounds. Cell Stem Cell 7, 651–655. 10.1016/j.stem.2010.11.015 21112560 PMC3812930

